# TORSEL, a 4EBP1-based mTORC1 live-cell sensor, reveals nutrient-sensing targeting by histone deacetylase inhibitors

**DOI:** 10.1186/s13578-024-01250-4

**Published:** 2024-06-01

**Authors:** Canrong Li, Yuguo Yi, Yingyi Ouyang, Fengzhi Chen, Chuxin Lu, Shujun Peng, Yifan Wang, Xinyu Chen, Xiao Yan, Haolun Xu, Shuiming Li, Lin Feng, Xiaoduo Xie

**Affiliations:** 1 School of Medicine, Shenzhen Campus of Sun Yat-sen University, Sun Yat-sen University, Shenzhen, China; 2 College of Life Sciences and Oceanography, Shenzhen Key Laboratory of Microbial Genetic Engineering, Shenzhen University, Shenzhen, China; 3 Department of Experimental Research, State Key Laboratory of Oncology in South China, Collaborative Innovation Center for Cancer Medicine, Sun Yat-sen University Cancer Center, Guangzhou, China

**Keywords:** mTORC1, Live-cell sensor, HDAC inhibitor, Panobinostat, Amino acid sensing

## Abstract

**Background:**

Mammalian or mechanistic target of rapamycin complex 1 (mTORC1) is an effective therapeutic target for diseases such as cancer, diabetes, aging, and neurodegeneration. However, an efficient tool for monitoring mTORC1 inhibition in living cells or tissues is lacking.

**Results:**

We developed a genetically encoded mTORC1 sensor called TORSEL. This sensor changes its fluorescence pattern from diffuse to punctate when 4EBP1 dephosphorylation occurs and interacts with eIF4E. TORSEL can specifically sense the physiological, pharmacological, and genetic inhibition of mTORC1 signaling in living cells and tissues. Importantly, TORSEL is a valuable tool for imaging-based visual screening of mTORC1 inhibitors. Using TORSEL, we identified histone deacetylase inhibitors that selectively block nutrient-sensing signaling to inhibit mTORC1.

**Conclusions:**

TORSEL is a unique living cell sensor that efficiently detects the inhibition of mTORC1 activity, and histone deacetylase inhibitors such as panobinostat target mTORC1 signaling through amino acid sensing.

**Supplementary Information:**

The online version contains supplementary material available at 10.1186/s13578-024-01250-4.

## Background

The highly conserved serine/threonine protein kinase mammalian or mechanistic target of rapamycin (mTOR) plays a central role in cell metabolism by coordinating cellular and extracellular signals such as growth factor (GF) and amino acid (AA) [1, 2]. Dysregulation of mTOR signaling is associated with human diseases including cancer, diabetes, aging, and mTORopathies [3, 4].

mTOR nucleates two structurally and functionally distinct mTOR complexes (mTORCs), namely, mTORC1 and mTORC2, to control cell growth and proliferation by phosphorylating substrates such as eukaryotic initiation factor 4E (eIF4E) binding protein 1 (4EBP1), ribosomal S6 kinase (S6K), and protein kinase B (PKB or AKT) [5]. Decades of research have established mTORC1 as a master regulator of cellular anabolic processes, including the synthesis of proteins, lipids, and other macromolecules, and of catabolic processes such as autophagy [2, 6]. mTORC1 is a lozenge-shaped dimer containing mTOR kinase, regulatory-associated protein of mTOR (RAPTOR), mammalian lethal with SEC13 protein 8 (mLST8), DEP domain-containing mTOR-interacting protein (DEPTOR), and proline-rich Akt substrate of 40 kDa (PRAS40) [4, 7]. Under nutrient-rich conditions, the Rag GTPases form heterodimeric complexes consisting of RagA/B bound to RagC/D. The complexes bind to mTORC1 and are tethered by the LAMTOR complex to the lysosomal surface, where mTORC1 is activated by the allosteric binding of Rheb [8–10]. GTP loading of RheB is essential for mTORC1 activation, which is negatively regulated by the upstream GTPase-activating protein (GAP) tuberous sclerosis complex (TSC) [11, 12]. The tumor suppressor TSC (TSC1/TSC2) integrates GF and stress-sensing signaling pathways including the PI3K/AKT, LKB1/AMPK, Wnt/GSK3, and ERK/RSK pathways, to regulate the interaction between RheB and mTORC1. Pathogenic mutations in these signaling pathways deregulate mTORC1 activity [3, 13]. AA sensing is another essential mechanism for mTORC1 activation, and essential amino acids (EAAs), such as leucine (Leu), arginine (Arg), and methionine (Met), are reported to bind to their sensor proteins to promote GTP loading of Rags via the inhibition of GATOR1 GAP activity [14]. Oncogenic mutations of GF signaling genes or AA-sensing genes such as PI3K, AKT, TSC, and GATOR1 lead to hyperactivation of mTORC1 and promote uncontrolled cell growth and malignant transformation [2, 13]. The inhibition of mTORC1 signaling has been shown to be clinically effective in human cancer therapy [5, 15].

Given the essential roles of mTORC1 in cell growth and its high relevance to various diseases, it is important to monitor the inhibition of mTORC1 in cells and exploit new inhibitors for precision medicine. Methods to detect mTORC1 activity are mostly immunochemical techniques based on antibodies recognizing specific phosphorylation sites of mTORC1 substrates, such as phospho-4EBP1 (T37/T46) antibodies, phospho-S6K1 (T389) antibodies, and phospho-S6 (S235/S236 or S240/S244) antibodies recognizing the downstream phosphorylated ribosomal protein S6. However, techniques such as immunoblotting (IB), immunofluorescence (IF), immunohistochemistry (IHC), and fluorescence-activated cell sorting (FACS) are strictly dependent on the specificity of these phosphor antibodies and require careful scaling to avoid background signals [16]; they typically require disruption or fixation of cells or tissues under nonphysiological conditions, and using antibodies for high-throughput screening is also expensive and time-consuming. To overcome such limitations, fluorescent kinase reporters for mTORC1 have been recently developed. For example, TORCAR and AIMTOR are genetically encoded reporters that allow noninvasive detection of mTORC1 activation by live-cell imaging [17, 18]. These reporters are valuable tools for studying mTORC1 kinase signaling at subcellular resolution and have recently been used to localize compartmentalized mTORC1 activity in the nucleus [19, 20]. However, the minor change in the fluorescence ratio between acceptor and donor fluorophores, and the substantial autofluorescence and light scattering effects limit their use in high-throughput drug screening and tissue applications [21]. In addition, TORCAR requires quantitative imaging of single cells, which limits its application to heterogeneous cell populations [17, 18]. Other kinase-dependent GFP translocation-based reporters (KTRs), such as GFP-LC3 or GFP-TFEB, which change their subcellular localization upon mTORC1 inactivation, are flawed because of either their low spatial resolution or nonspecific responses [22]. Therefore, alternative fluorescence reporters are needed to visualize mTORC1 activity in living cells and tissues. Here, we developed a live-cell mTORC1 sensor (TORSEL) with simple manipulation and a high-contrast signal pattern to specifically detect mTORC1 inhibition in cultured cells and tissues.

Histone deacetylases (HDACs) remove acetyl groups from acetyl-lysine residues in histones and non-histone proteins and play important roles in a variety of biological processes through epigenetic modification and gene transcription regulation [23, 24]. Histone deacetylase inhibitors (HDACis), such as panobinostat and entinostat, have been approved for the treatment of hematologic malignancies with broad target specificity via multiple mechanisms, including the induction of cell death, cell cycle arrest, apoptosis, differentiation, and the promotion of immunogenicity [23, 24]. Nonetheless, little is known about how HDACis affect critical metabolic signaling pathways such as mTORC1. In this study, we used TORSEL to visually screen drugs that can inhibit mTORC1 in living cells. Our findings show that HDACis strongly inhibit mTORC1 in vitro and in vivo. Further mechanistic studies revealed that HDACis have a unique mechanism of action (MOA) that targets the amino acid-sensing pathway.

## Results

### Design and characterization of TORSEL

**Fig. 1:**
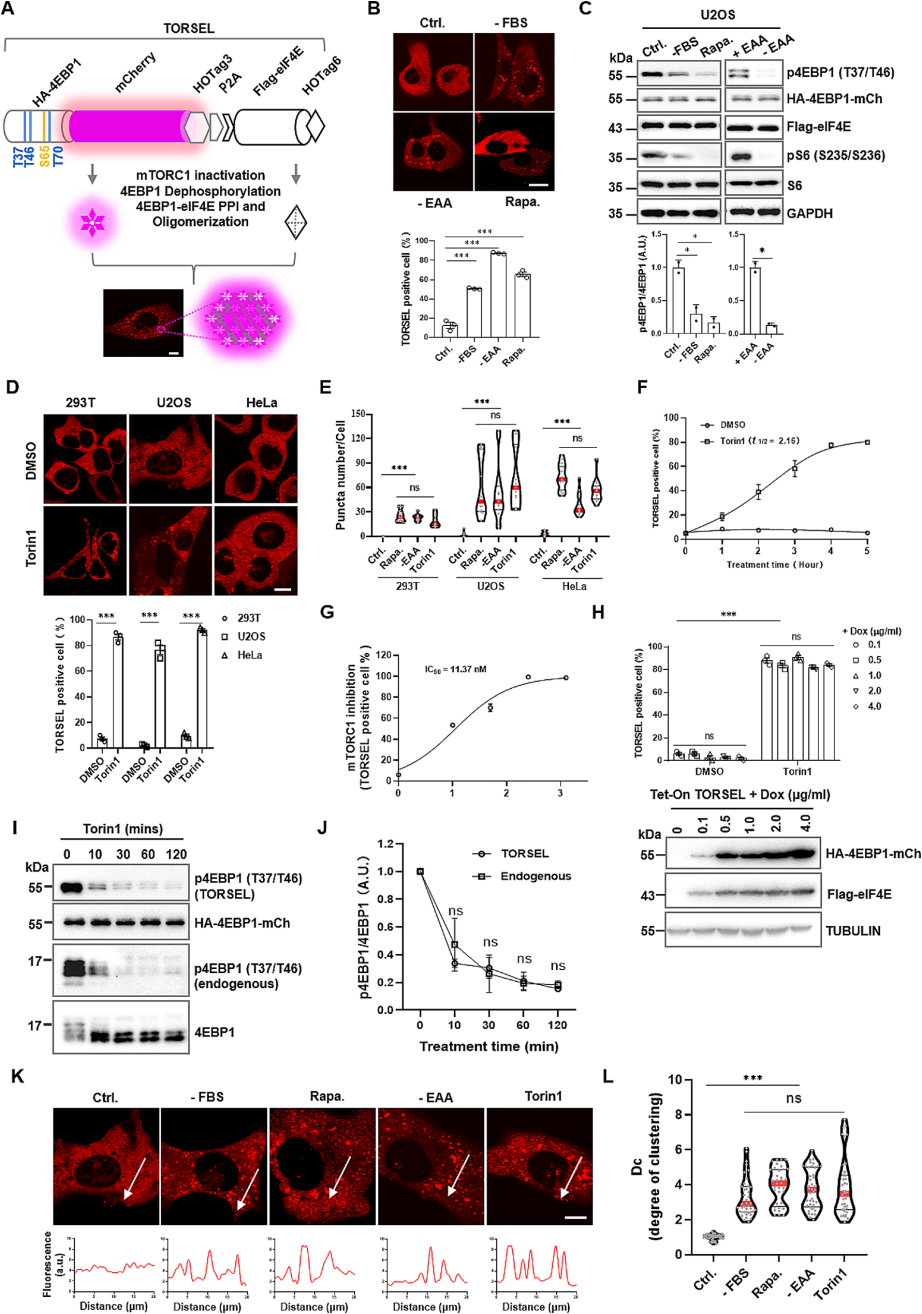
Design and characterization of TORSEL. (**A**) Schematic diagram of TORSEL puncta formation. (**B**) Representative images of TORSEL in U2OS cells (100 nM rapamycin, EAA or FBS starvation 12 h) (upper panel) and quantification of TORSEL-positive cells (defined as > 10 puncta/cell) (lower panel). (**C**) IB analysis of TORSEL-transfected U2OS cells (upper panel) and quantification of 4EBP1 (T37/T46) phosphorylation by signal ratios normalized to those of the untreated control (= 1.0) (lower panel). (**D**) Representative images of TORSEL in cell lines treated with Torin1 (50 nM, 12 h) (upper panel) and quantification of TORSEL-positive cells (lower panel). (**E**) Violin plots of puncta size in cell lines with various treatments. Red line, median; black line, interquartile range; 15–20 cells were analyzed for each sample. (**F**) Time-response curve of TORSEL in U2OS cells treated with Torin1. *t*_*1/2*_ were calculated with nonlinear fit analysis. (**G**) Dose-response curve of TORSEL treated with various doses of Torin1 for 12 h. The *IC50* was calculated with nonlinear fit analysis. (**H**) Tet-ON TORSEL responses in 293T cells induced by dose-escalating Dox (lower panel), and were quantified for each dose (upper panel). (**I**) IB analysis of 4EBP1 dephosphorylation kinetics in response to Torin1 in 293T cells. The experiment was performed twice. (**J**) Quantified 4EBP1 dephosphorylation kinetics in (**I**). The p4EBP1/4EBP1 ratio was normalized to 1 at time point zero. (**K**) Representative images of TORSEL puncta in U2OS cells with various treatments; the fluorescence histograms are plotted with lines across the cells. (**L**) Violin plots of Dc values in U2OS cells with different treatments (*n* = 50 puncta). the Data are presented as the mean ± SEM. Approximately 50 cells in each sample were calculated by the percentage of TORSEL-positive cells. Scale bar, 10 μm. Statistical analysis was performed using two-tailed unpaired Student’s t-test. ns, no statistical significance, ^*^P < 0.05, ^**^P < 0.01, ^***^P < 0.001

To directly visualize the inactivation of mTORC1 in living cells, we created a synthetic fluorescent biosensor called TORSEL (m*TOR*C1 *se*nsor for *l*ive cells). TORSEL comprises two parts: HA-tagged 4EBP1, which is tagged with the fluorescent protein mCherry and homohexameric tag 3 (HOTag3); and Flag-eIF4E, which is tagged with homotetrameric tag 6 (HOTag6) [25, 26]. The two pieces are linked by a self-cleaving P2A peptide (Fig. 1A). The 17-kDa 4EBP1 is a high-quality mTORC1 substrate that binds strongly to the 25-kDa eukaryotic initiation factor 4E (eIF4E) in its dephosphorylated form [27, 28]. To initiate cap-dependent mRNA translation, mTORC1 hierarchically phosphorylates 4EBP1 at four major sites, Thr37, Thr46, Ser65, and Thr70, which free eIF4E from the tightly bound 4EBP1-eIF4E complex to form the translation initiation complex [29–31] (Fig. S1A). Under normal culture conditions, TORSEL is expressed diffusely in U2OS cells. When mTORC1 is suppressed by serum deprivation, EAA starvation, or rapamycin, 4EBP1 within TORSEL undergoes dephosphorylation; once dephosphorylated, 4EBP1 interacts with eIF4E and connects the two components of TORSEL. This protein-protein interaction (PPI), along with HOTag polymerization in each part, causes a multiplex PPI that creates bright fluorescent puncta similar to those of the SPPIER and SPARK reporters [25, 26]. Most cells (50-90%) showed visible puncta patterns under a fluorescence microscope with a 60x oil objective (Fig. 1A, B and C and Fig. S1B). The TORSEL response parameters were quantified in various cell lines treated with the mTOR kinase inhibitor Torin1, which induced a high percentage of puncta cells (70-90%) (Fig. 1D); the number of puncta (10–100 per cell) dramatically increased with different mTOR inhibitors in each cell line (Fig. 1E). TORSEL responded to Torin1 with a half-maximum inhibition time (*t*_*1/2*_) of 2.16 h and a half-maximum inhibitory concentration (*IC*_*50*_) of 11.37 nM in U2OS cells (Fig. 1F and G). It is less sensitive in 293T cells compared to U2OS cells (Fig. S1C). These results suggest that TORSEL exhibits different sensitivities in different cells. The response of TORSEL to mTORC1 inhibition is independent of TORSEL expression levels. In stable clones of 293T reporter cells, a high TORSEL expression level resulted in a slight increase in the number of background puncta, but the percentage of cells with TORSEL puncta remained more than 5 times greater in response to Torin1 across all of the tested clones (Fig. S1D). We also observed a consistent response of TORSEL with increased expression levels induced by escalating doses of doxycycline (Dox) using an inducible Tet-On TORSEL (Fig. 1H). These findings suggest that the expression level of TORSEL does not impact its responsiveness. In addition, identical 4EBP1 dephosphorylation kinetics were observed in response to Torin1 treatment for TORSEL and endogenous 4EBP1 (Fig. 1I and J), which suggests that TORSEL authentically reflects the inhibition of cellular mTORC1 activity. The fluorescent puncta were quantified and plotted as a fluorescence histogram at the pixel level, showing high contrast in response to mTORC1 inhibition (Fig. 1K). The degree of clustering (Dc) was quantified by the ratio of fluorescence intensity between the puncta and the nearby cytosol [32]. Dc values in mTORC1-inhibited cells were 2–4 times greater than those in untreated cells (Fig. 1L), which suggested a relatively high resolution of the reporter.

### TORSEL responds to mTORC1-mediated 4EBP1 phosphorylation in living cells and tissues

**Fig. 2:**
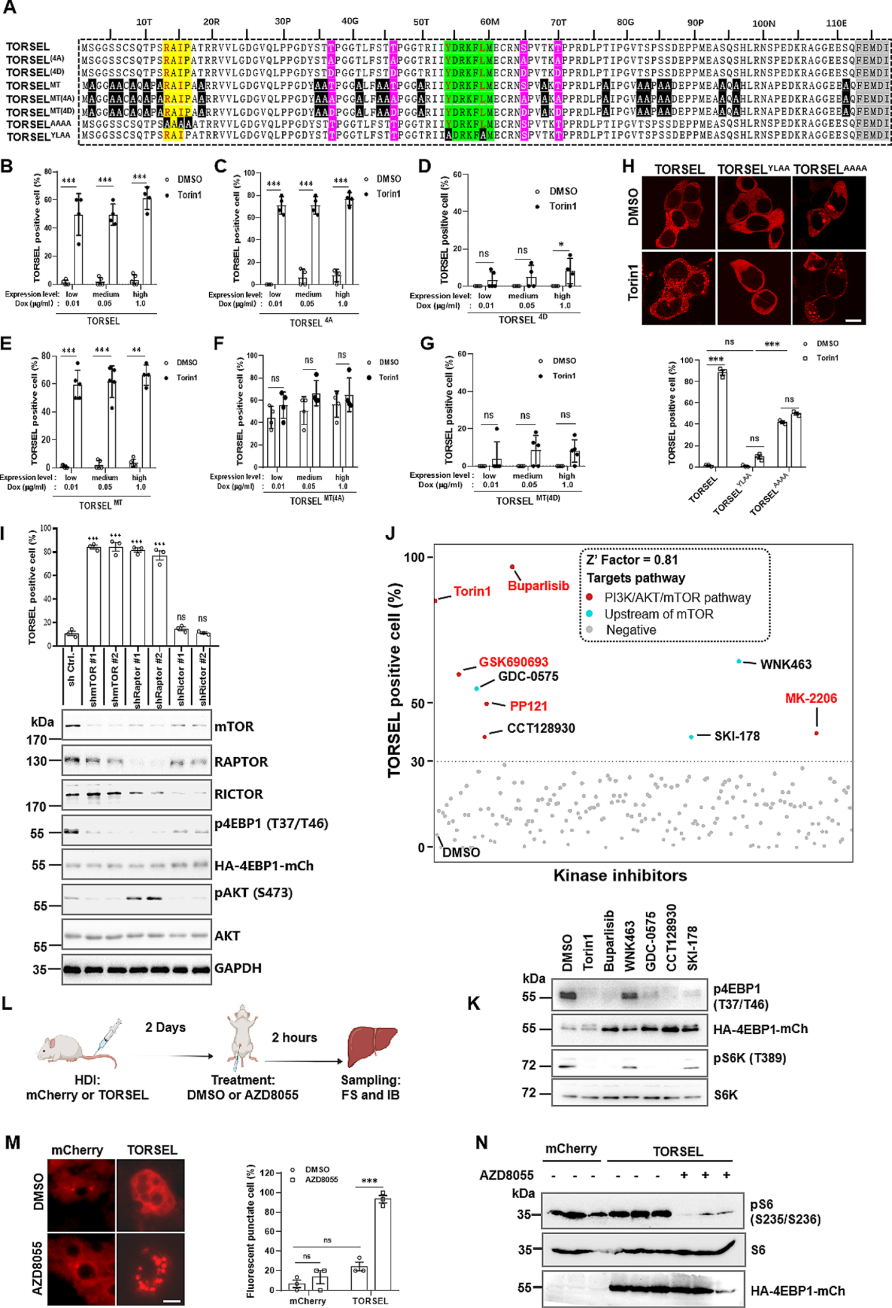
TORSEL specifically responded to mTORC1-mediated 4EBP1 phosphorylation. (**A**) Aligned sequences of 4EBP1 and mutants in TORSEL. The major mTOR sites are in pink; the sites mutated to Ala are in black; RAIP in yellow, and YxxxxLΦ in green. (**B**)-(**G**) Responses of TORSELs to Torin1 at different expression levels. TORSELs were induced by dose-escalating Dox (0.01, 0.05, or 1.0 µg/ml). The mean fluorescence intensities (MFIs) at low, medium, and high expression levels for TORSEL were 19.6 ± 11.22, 74.08 ± 18.5, and 148.24 ± 22.18 (**B**); for TORSEL^4A^, 20.14 ± 7.25, 52.64 ± 8.93, and 119.49 ± 8.88 (**C**); for TORSEL^4D^, 24.6 ± 4.68, 57.35 ± 9.57, and 134.14 ± 37.49 (**D**); for TORSEL^MT^, 12.84 ± 6.83, 70.46 ± 7.29, and 116.95 ± 20.93 (**E**); for TORSEL^MT(4A)^, 18.71 ± 8.58 and 70.18 ± 18.8 and 144.13 ± 18.95 (**F**); and for TORSEL^MT(4D)^, 21.84 ± 3.77 and 69.15 ± 21.11 and 117.03 ± 22.14 (**G**). (**H**) Responses of TORSEL^AAAA^ and TORSEL^YLAA^ to Torin1 (50 nM, 12 h) in 293T cells. (**I**) TORSEL responses and IB analysis of mTORC subunit-knockdown 293T cells. (**J**) Scatter plots of kinase library screening by TORSEL. The red-highlighted inhibitors inhibit mTORC1; red dots denote known PI3K/AKT/mTOR inhibitors; cyan dots denote inhibitors that target mTORC1 upstream signaling as referenced in published literature summarized in Table S4; gray dots indicate no or weak inhibitory activity; and the dotted line shows the 30% threshold for screening. (**K**) IB validation of mTORC1 inhibition by positive hits targeting mTORC1 upstream in 293T cells. (**L**) Flow diagram of TORSEL delivery in mouse liver tissue (HDI: hydrodynamic injection, FS: frozen sectioning). (**M**) Representative images from frozen sections of mouse livers expressing TORSEL (left) and quantified punctate cells (right). Approximately 50 cells were calculated in each of the 3 liver samples. (**N**) IB analysis of mouse liver tissues (*n* = 3). The Data are presented as the mean ± SEM. About 50 cells were calculated by percentage of TORSEL-positive cells. Scale bar, 10 μm. Two-tailed unpaired Student’s t-test were used for statistical analysis; ns, no statistical significance, ^*^P < 0.05, ^**^P < 0.01, ^***^P < 0.001

To determine whether TORSEL puncta formation is determined by the specific regulation of 4EBP1 phosphorylation by mTORC1, phosphomimetic and nonphosphorylatable mutants of 4EBP1 with corresponding mutations in TORSEL at four major mTORC1 sites (Thr37, Thr46, Ser65, and Thr70) were generated and expressed by the Tet-On expression system [33, 34] (Fig. 2A, Table S1). We induced reporter expression at three levels (low, medium, and high, grouped by mean fluorescence intensity) in 293T cells by escalating doses of doxycycline (Dox), as shown in Fig. 1H and Fig. S2A. Then, we quantified the TORSEL responses in single cells with different fluorescence intensities that were directly correlated with the intracellular protein concentration [35] (Fig. 2B–G). The TORSEL^(4D)^ mutant with four mTORC1 phosphorylation sites that were mutated to Asp no longer responded to Torin1 except for a mild response with a high expression level (Fig. 2D); however, the TORSEL^(4 A)^ mutant with four mTORC1 phosphorylation sites that were mutated to Ala continued to respond to Torin1 at all levels (Fig. 2C); this effect could be due to extra mTORC1-regulated sites (either directly or indirectly). To test this possibility, we generated TORSEL^MT^ with all 22 Thr/Ser sites mutated to Ala with the exception of four mTORC1 sites (Fig. 2A). This mutant responded to mTORC1 inhibition in the same way as wild-type TORSEL at all expression levels (Fig. 2B and E). Notably, TORSEL^MT(4 A)^ or TORSEL^MT(4D)^ with all mutated phosphorylation sites abolished the response to Torin1, whereas TORSEL^MT(4 A)^ constitutively formed puncta with or without mTORC1 inhibition (Fig. 2F and G). Similar results were also obtained in U2OS cells with regular expression vectors (Fig. S2B). The discrepancy between TORSEL^(4 A)^ and TORSEL^MT(4 A)^ suggests that in addition to the four major phosphorylation sites of 4EBP1, there are other potential sites regulated by mTORC1 that affect the formation of TORSEL puncta. We then compared TORSEL with TORSEL^MT^ and revealed that both responded similarly to various mTORC1 inhibitions in different cell lines including U2OS, MCF7, LO2, A549, and mouse embryonic fibroblasts (MEFs) (Fig. S2C, S2D). To reiterate the specificity of TORSEL in response to 4EBP1 phosphorylation by mTORC1, we generated a TORSEL^AAAA^ mutant in which the RAIP motif was mutated to AAAA. This mutant could not be phosphorylated since RAIP is an anchor site for mTORC1 [36, 37]. Indeed, we observed constitutive puncta formation of the TORSEL^AAAA^ mutant with or without mTORC1 inhibition, while no puncta formation was observed for the YLAA (TORSEL^YLAA^) mutant even with Torin1 treatment (Y and L mutated to A and rendered the eIF4E binding motif “YxxxxLΦ” dysfunctional) [38] (Fig. 2A and H). These results indicate that TORSEL can effectively respond to mTORC1 inhibition through mTORC1-specific 4EBP1 phosphorylation. Additionally, TORSEL responded to RAPTOR or mTOR depletion but not to RICTOR depletion by shRNA knockdown (Fig. 2I), which suggested that TORSEL specifically responded to mTORC1 but not to mTORC2 deficiency. The GAP protein GATOR1 inhibits Rag GTPases in response to EAA starvation; sgRNA targeting DEPDC5, one of the subunits of GATOR1, blocked 4EBP1 dephosphorylation and the TORSEL responses induced by EAA starvation (Fig. S2E-S2G). These results indicate that TORSEL is a specific tool for visualizing genetic, pharmacological and physiological inhibition of mTORC1.

To further clarify the specificity of TORSEL, we screened a kinase inhibitor library containing 212 validated inhibitors of 103 frequently targeted protein kinases (Table S2), including those reported as mTOR-dependent or mTOR-independent 4EBP1 kinases such as AKT, CDK1, and GSK3 [39–42] (Table S3). As expected, TORSEL specifically responded to inhibitors targeting PI3K/AKT/mTORC1 signaling (5 of 8 positive hits) or related upstream regulating kinases (3 of 8 positive hits) (Fig. 2J). Among them, PP121, MK-2206, GSK690693, and Buparlisib have previously been reported to inhibit mTORC1 (Table S4), which validated that TORSEL screening was effective. Other hits, such as GDC-0575 (CHK1 inhibitor), SKI-178 (SPHK inhibitor), CCT128930 (AKT inhibitor), and WNK464 (WNK inhibitor), similarly inhibited mTORC1 via upstream signaling, as previously reported (Table S4). The inhibition of mTORC1 by these inhibitors was also validated by immunoblotting (Fig. 2K). Further examination of the kinase inhibitor profiling data revealed that TORSEL did not respond to any of the mTOR-independent 4EBP1 kinases (Fig. S2H). Additionally, the effects of individual inhibitors targeting putative 4EBP1 kinases were compared using TORSEL and immunoblotting. AKT and MEK (mTOR-dependent 4EBP1 kinase) inhibitors and various mTORC1 inhibitors activated TORSEL with dephosphorylated 4EBP1 (Fig. S2I). We did not detect TORSEL puncta formation or 4EBP1 dephosphorylation in response to inhibitors targeting putative mTOR-independent 4EBP1 kinases, such as CDK1, GSK3, ATM, or ATR, under normal culture conditions (Fig. S2I), as these kinases may require a specific precondition to phosphorylate 4EBP1. Hence, mTORC1 is the primary kinase responsible for 4EBP1 phosphorylation in normal cultures, and TORSEL responds specifically to mTORC1 inhibition.

TORCAR is a FRET-based mTORC1 sensor that reports mTORC1 activity upon GF stimulation by FRET signals at the single-cell level [17] (Fig. S2J). However, neither insulin stimulation nor Torin1 inhibition caused FRET signal changes in 96-well plated cell population (Fig. S2K, S2L). These data suggest that TORCAR could not be used in plate-reading mode for high-throughput screening. In contrast, TORSEL kinase inhibitor screening showed a Z’ factor of 0.81, which suggests that TORSEL has excellent performance in living cell screening (Fig. 2J). TORSEL also demonstrated a substantially stronger response (2-4-fold change in Dc values) in response to Torin1 than TORCAR at the single-cell level [43] (Fig. S2M). Therefore, TORSEL could be preferentially applied as a high-throughput screening tool in plate-reading mode. The poor resolution and high background have limited the application of fluorescence sensors, such as TORCAR or AIMTOR, for reporting mTORC1 activity in mouse tissue. To test whether TORSEL could report the mTORC1 inhibition in tissue, we expressed mTORC1 in the mouse liver by hydrodynamic injection (Fig. 2L). Cryosection imaging revealed that TORSEL was expressed in liver tissue in a diffuse pattern, and it was converted to bright puncta in mice subcutaneously injected with the mTOR kinase inhibitor AZD8055 (Fig. 2M and N). These data suggest that the formation of TORSEL puncta is a 4EBP1 dephosphorylation-specific event that occurs in response to mTORC1 inhibition both in vitro and in vivo.

### TORSEL is modulated by mTORC1 kinase and protein phosphatase

**Fig. 3:**
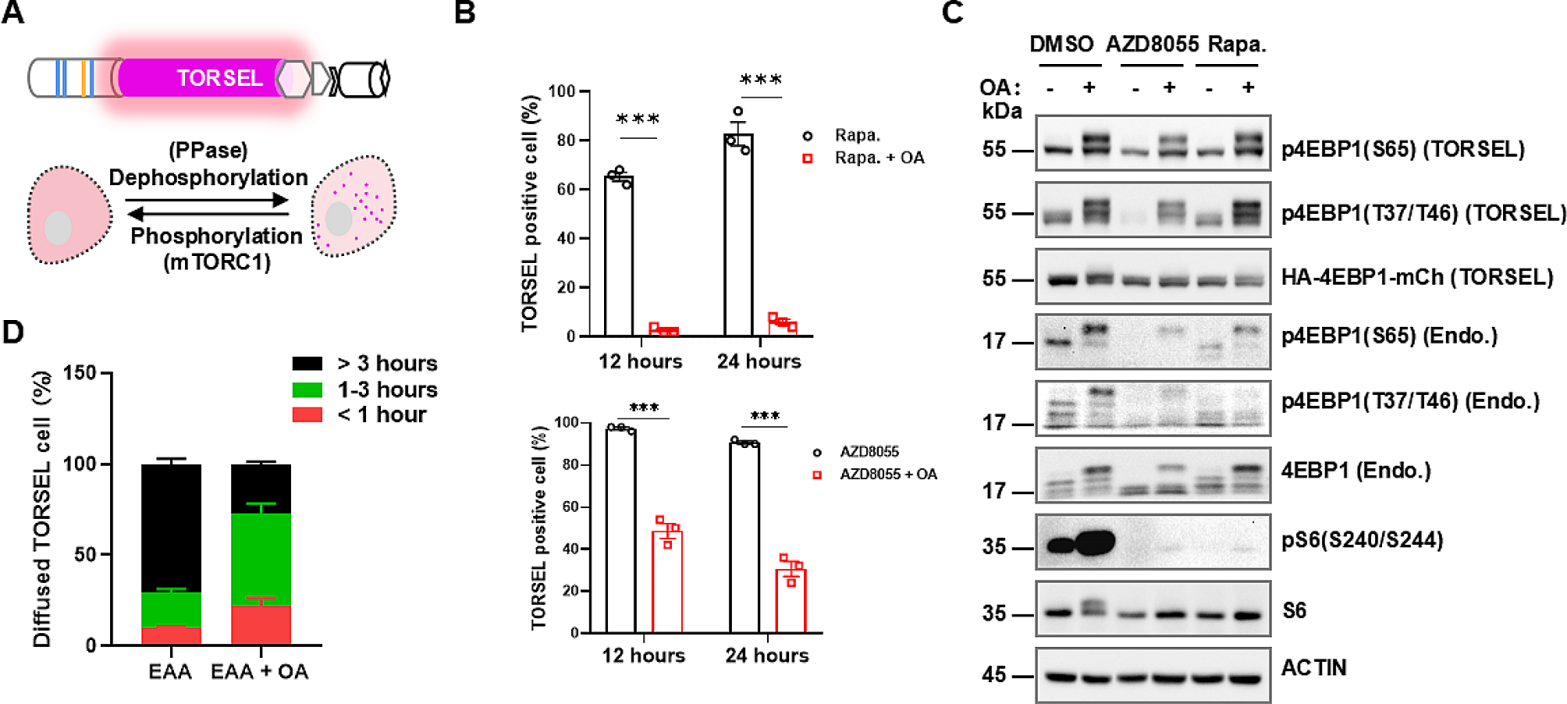
Modulation of TORSEL by protein phosphatases. (**A**) Diagram of TORSEL regulation by mTORC1 and protein phosphatases. (**B**) Effect of OA on TORSEL puncta formation induced by rapamycin (upper panel) and AZD8055 (lower panel) in 293T cells. (**C**) IB analysis of the effect of OA on 4EBP1 phosphorylation in response to 100 nM rapamycin or 100 nM AZD8055. (**D**) TORSEL puncta diffusion analysis upon refeeding of EAA with or without 20 nM OA pretreatment. 293T cells were EAA starved of EAA for 12 h to induce TORSEL puncta, then, EAA or EAA + OA (20 nM) was added, and the percentage of TORSEL-positive cells was analyzed by imaging at regular intervals for 24 h. The Data are presented as the mean ± SEM. Approximately 50 cells in each sample were calculated as the percentage of TORSEL-positive cells; statistical analysis was performed using two-tailed unpaired Student’s t-test. ^***^P < 0.001

The interaction between 4EBP1 and eIF4E is determined by 4EBP1 phosphorylation status, which is balanced by the mTORC1 kinase and the 4EBP1 phosphatase [27, 44] (Fig. 3A). To determine whether phosphatases play roles in the TORSEL response, we applied okadaic acid (OA) to inhibit PP1/PP2A, which are known 4EBP1 protein phosphatases [45, 46]. Indeed, OA efficiently blocked the formation of puncta induced by AZD8055 or rapamycin (Fig. 3B), and biochemical data verified its effects on 4EBP1 phosphorylation (Fig. 3C). These results confirm that 4EBP1 dephosphorylation by phosphatases is essential for TORSEL to respond to mTORC1 inhibition. AA restimulation of mTORC1 did not immediately disperse all of the TORSEL puncta induced by EAA starvation; only approximately 10% of the punctate cells diffused within 1 h, 30% of them diffused within 3 h, and 70% of them persisted for more than 3 h (Fig. 3D). This slow reversibility could be attributed to the biophysical properties of the TORSEL puncta, which could not fully recover from the fluorescence recovery after photobleaching (FRAP) experiment (Fig. S3A, S3B). Additionally, the puncta were insensitive to the protein droplet disperser 1,6-hexanediol (HEX) (Fig. S3C). These data suggest that TORSEL does not behave as the SPARK or SPPIER reporters in reversibility [25, 26, 47]. To determine whether these less reversible puncta are toxic to cells, we generated stable 293T cell lines with mCherry, diffuse TORSEL (TORSEL, TORSEL^MT^, and TORSEL^MT(4D)^) and constitutive punctate TORSEL (TORSEL^AAAA^ and TORSEL^MT(4 A)^ ); both diffuse and punctate reporter cells proliferated normally in one week, and no obvious cytotoxicity was observed (Fig. S3D). To test whether phosphatases are involved in TORSEL puncta diffusion, OA and CdCl2 were utilized to inhibit the phosphatases PP1/PP2A and phosphatase PPM1G, respectively [45, 46]. OA treatment promoted the diffusion of up to 75% of the puncta cells within 3 h, although 25% of them remained puncta positive (Fig. 3D). OA also prevented puncta formation induced by the AKT inhibitor GSK690693 and promoted puncta diffusion after drug washout (Fig. S3E, S3F). The PPM1G inhibitor CdCl2 did not prevent puncta formation alone but promoted puncta diffusion as did OA (Fig. S3E, S3F), which implies that different types of phosphatases may affect the TORSEL response differently. TORSEL is localized in the cytosol, and we also tested whether cell organelle-located TORSELs could respond to Torin1 inhibition at specific subcellular sites, such as the mitochondria (Mit-TORSEL), plasma membrane (PM-TORSEL), endoplasmic reticulum (ER-TORSEL), and lysosome (Lyso-TORSEL), but none of them showed the same response as TORSEL (Fig. S3G). Overall, these results suggest that TORSEL is regulated by mTORC1 and phosphatases in the cytosol but has slow kinetics in terms of response speed and reversibility; therefore, it may not represent the real-time kinetics of physiological mTORC1 regulation but rather regulation over prolonged time.

### TORSEL live-cell screening identified HDACis as mTORC1 inhibitors targeting GATOR1-mediated AA signaling

**Fig. 4:**
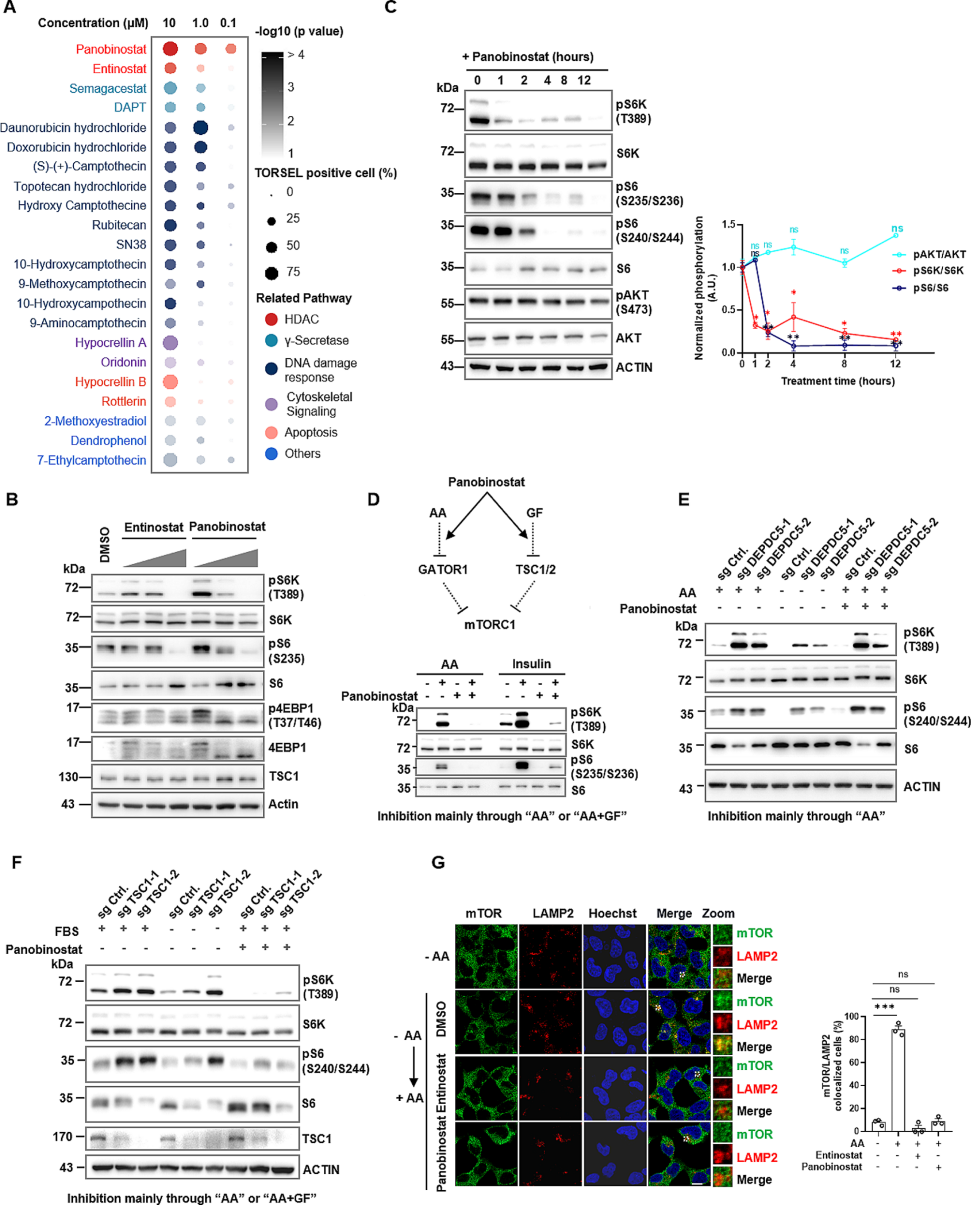
TORSEL live-cell screening identified nutrient-sensing targets for panobinostat. (**A**) Bubble plot of dose-dependent responses of TORSEL to screened positive hits in 293T cells. (**B**) IB analysis of mTORC1 signaling in 293T cells treated with various doses of HDACis (0.1, 1, or 10 µM for 12 h). (**C**) IB analysis of mTORC1 and mTORC2 activity in response to panobinostat. 293T cells were treated with 10 µM panobinostat for 12 h, and mTOR activities were quantified by pS6K/S6K, pS6/S6, and pAKT/AKT ratios. Two independent experiments were performed. (**D**) Diagram of possible pathways for mTORC1 inhibition by panobinostat (upper panel) and IB analysis of mTORC1 activity in response to panobinostat combined with AA or insulin stimulation (lower panel). 293T cells were pretreated with DMSO or 10 µM panobinostat for 12 h, starved of AA for 50 min and restimulated with AA for 15 min, or serum starved for 12 h and restimulated with 100 nM insulin for 15 min. (**E**) IB analysis of mTORC1 activity after panobinostat treatment in DEPDC5-deficient 293T cells with or without AA stimulation. (**F**) IB analysis of mTORC1 activity in TSC1-deficient 293T cells treated with or without FBS stimulation and panobinostat inhibition. (**G**) The effect of HDACis on mTOR and lysosomal colocalization. 293T cells were pretreated with HDACis for 12 h and then starved and stimulated with AA as indicated. IF was performed with mTOR and LAMP2 antibodies, and the cells were imaged by confocal microscopy (left) and quantified by counting the percentage of cells costained with mTOR and LAMP2 (right), *n* = 50 cells. The Data are presented as the mean ± SEM. Scale bar, 10 μm. Statistical analysis was performed using two-tailed unpaired Student’s t-test. ns, no statistical significance, ^*^P < 0.05, ^**^P < 0.01, ^***^P < 0.001

mTOR inhibitors are therapeutically promising for targeted therapy of cancer and other mTOR-related diseases [48, 49]. Using TORSEL-expressing 293T cells, we visually screened a drug library containing 917 FDA-approved drugs and 1059 natural antitumor ingredients (Fig. S4A). We found several clusters of drugs, including DNA damage agents, Ƴ-secretase inhibitors, and histone deacetylase inhibitors (HDACis), that had notable puncta-promoting effects on TORSEL (Fig. 4A, Fig. S4B, and Table S4). Most hits were DNA damage agents that inhibit DNA topoisomerases; these compounds are known to inhibit mTORC1 signaling through the p53-AMPK-TSC pathway [50]. Other hits, such as rottlerin and oridonin, have been reported as mTORC1 signaling inhibitors [51–53], which validated the effectiveness of this screening (Table S4). Positive hits were further selectively validated by immunoblotting (Fig. S4C), and the most potent hits, HDACi panobinostat and entinostat, blocked mTORC1 activity both in 293T cells and in MCF7 cells (Fig. 4B, Fig. S4D). To determine whether other HDACis have general inhibitory effects on mTORC1, we tested seven HDACis, namely, SAHA, TSA, panobinostat, VPA, chidamide, romidepsin, and entinostat, across three cell lines. Our results showed that mTORC1 was generally inhibited, although certain inhibitors did not affect specific cell types (Fig. S4E, S4F). For example, panobinostat, a pan-HDACi, inhibited mTORC1 in all three tested cell lines. SAHA and romidepsin had no effect on 293T cells, while VPA and chidamide had little effect on MCF7 cells. Similar to Torin1, panobinostat and entinostat induced significant autophagy as indicated by LC3 puncta induction, which is a commonly documented effect of HDACis [54, 55] (Fig. S4G). These results suggest that HDACis have a general inhibitory effect on mTORC1, although the effects may differ depending on the cellular context.

Several studies have reported that some HDACis, such as TSA and SAHA, can affect AKT (S473) phosphorylation or TSC1 expression [56, 57]; however, no change in TSC1 expression or AKT (S473) phosphorylation was observed in our experiments (Fig. 4C, Fig. S4D, S4H), which suggests that these HDACis specifically inhibit mTORC1 but not mTORC2. There are three possible ways for HDACis to suppress mTORC1 signaling, as illustrated in Fig. 4D (upper panel): “AA” (AA signaling), “GF” (GF signaling), and “AA + GF” (both pathways). Panobinostat nearly blocked mTORC1 activity upon AA restimulation but only partially inhibited mTORC1 activity upon insulin stimulation (Fig. 4D, lower panel). In this case, panobinostat inhibits mTORC1 mainly through “AA” or “AA + GF” but less likely through “GF”. Constitutively active mTORC1 in GATOR1-deficient cells cannot be inhibited by panobinostat under AA stimulation conditions, which suggests that this inhibition requires GATOR1 and mainly acts through “AA” rather than “GF” (Fig. 4E). Panobinostat can inhibit GF-induced mTORC1 activation in TSC1-deficient cells, which suggests that panobinostat acts through “AA” or “AA + GF” but less likely through TSC1-mediated “GF” (Fig. 4F). Similar results were obtained for entinostat (Fig. S4H, S4I). Additionally, both HDACis inhibited mTOR translocation to lysosomes in response to AA refeeding as indicated by the loss of mTOR and LAMP2 colocalization (Fig. 4G). Collectively, these data indicate that panobinostat inhibits mTORC1 mainly through GATOR1-mediated AA signaling.

### Transcriptional induction of AA sensors by HDACis contributes to the inhibition of mTORC1

**Fig. 5:**
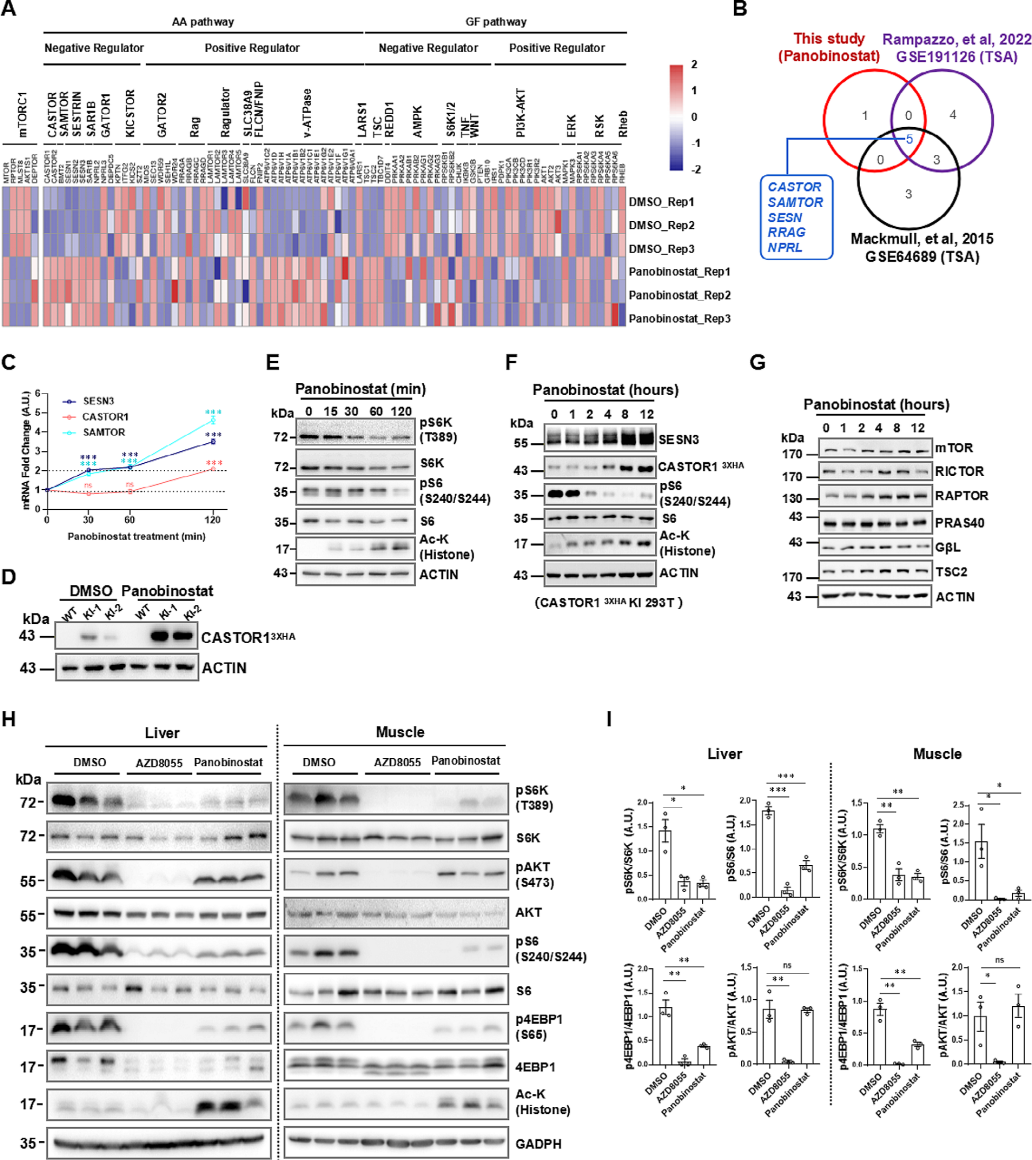
AA sensor induction by HDACis contributes to mTORC1 inhibition. (**A**) Gene expression profiling of panobinostat-treated 293T cells. Heatmap analysis of differentially expressed genes (DEGs) in panobinostat-treated (10 µM, 12 h) 293T cells by high-throughput RNA-seq. The DEGs were grouped by positive and negative regulators of mTORC1 in the AA or GF signaling pathway. The color scale (− 2.0 to 2.0) represents the calculated Z score. (**B**) Venn diagram showing the DEGs shared by the three transcriptomic datasets in the AA pathway. (**C**) Time-course induction of AA sensor mRNA by panobinostat (10 µM) in 293T cells. (**D**) Induction of endogenously tagged CASTOR1^3XHA^ by panobinostat (10 µM, 12 h) in 293T cells. The endogenous tagging of CASTOR1 is described in the methods section. (**E**) Two-hour time course of mTORC1 inhibition by panobinostat in 293T cells. (**F**) Time course induction of AA sensor proteins by panobinostat in CASTOR1 ^3XHA^-KI 293T cells. (**G**) Analysis of mTORC1 subunit expression by panobinostat treatment in 293T cells. (**H**) The IB analysis of mTOR signaling in mouse liver and muscle tissues treated with panobinostat; samples from 3 mice per group for each treatment. (**I**) The quantifications of mTOR activity (pS6K, pS6, p4EBP1, and pAKT were normalized to total S6K, S6, 4EBP1, and AKT protein levels, respectively) inhibited by panobinostat and Torin1 in mouse liver and muscle tissues in (H); the data were then normalized to the DMSO-treated control. The Data are presented as the mean ± SEM. Scale bar, 10 μm. Statistical analysis was performed using two-tailed unpaired Student’s t-test. ns, no statistical significance, ^*^P < 0.05, ^**^P < 0.01, ^***^P < 0.001

HDACs are key epigenetic regulators that control gene expression in cells. To explore the underlying mechanisms of HDACis in mTORC1 inhibition, we performed transcriptomic RNA sequencing (RNA-seq) to profile whole-genome expression changes upon panobinostat treatment in 293T cells. We selected 89 genes in the mTOR KEGG pathway as a reference to analyze transcriptomic data and identify mTORC1 upstream targets of panobinostat (Table S5). Analysis of RNA sequencing data from our study and two previously published microarray datasets (GSE191126 and GSE64689 [58, 59]) revealed that the mRNA expression levels of amino acid sensor genes such as *CASTOR*, *SAMTOR*, and *SESTRIN* were consistently up-regulated across all three datasets, while *RagA/C* and *NPRL2* were up-regulated and *RagB/D* and *NPRL3* were down-regulated in the datasets (Fig. 5A and B, and Fig. S5A). To verify the sequencing data, we treated three cell lines with four HDACis and measured the mRNA levels of major mTORC1 inhibitory genes, such as AA sensor genes and GATOR1 subunit genes involved in AA-sensing signaling, as well as genes downstream of GF and stress signaling, such as *TSC1*, *TSC2*, *REDD1*, and *PTEN* (Fig. S5B). HDACis consistently increased the transcripts of AA sensor genes, including *CASTOR1* (*CASTOR2* in MCF7), *SAMTOR*, and *SESTRIN3*, in all cell lines (at least a 2-fold change induced by 3 of 4 HDACis). Additionally, *SESTRIN3* mRNA levels were dramatically induced (Fig. S5B) as previously reported [60, 61]. In contrast, only *PTEN* mRNA levels were mildly induced by HDACi treatment in 293T cells, whereas *TSC1* or *TSC2* exhibited negligible changes under all conditions tested (Fig. S5B), which is consistent with the immunoblotting results (TSC1 in Fig. 4B and Fig. S4D).

Panobinostat completely prevents the activation of mTORC1 through the sensors SESTRIN3, CASTOR1, and SAMTOR via single amino acid (sAA) stimulation of Leu, Arg, or Met (Fig. S5C). *SAMTOR* and *SESTRIN3* mRNA induction was evident at 30 min, preceding *CASTOR1* induction at 2 h, which indicated sequential induction of the sensor genes (Fig. 5C); additionally, panobinostat was found to induce the expression of the endogenously tagged CASTOR1^3xHA^, SESTRIN3, TSC2, and Raptor proteins but not the other subunits of mTORC1 (Fig. 5D, F and G and Fig. S5D). We carefully examined the time course of mTORC1 inhibition by panobinostat and found that mTORC1 was partially inhibited before the 2-hour treatment, as evidenced by the partial decrease in pS6K and pS6 (Figs. 4C and 5E). However, there was no significant increase in the SENS3 or CASTOR1 protein before the 2-hour treatment. SENS3 or CASTOR1 protein induction was initially observed at approximately 4 h and peaked at 8–12 h (Fig. 5F). Moreover, pS6K or pS6 were fully inhibited after 4 h (Figs. 4C and 5F). These findings suggest that other mechanisms, in addition to the transcriptional regulation of AA sensors, might also be involved in the inhibition of AA sensing, particularly in the early stages of inhibition. Notably, panobinostat likely inhibits mTORC1 via the combined effects of multiple AA sensors, as single knockouts of either CASTOR1 or SAMTOR did not abolish this inhibition; double knockouts of both sensors alleviated the inhibitory effects (Fig. S5E-S5H). This result indicated that other sensors or mechanisms could also contribute to the inhibition of mTORC1 by panobinostat. Finally, we examined the physiological effects of panobinostat in mouse tissues. Compared to the kinase inhibitor AZD8055, panobinostat specifically inhibited mTORC1 without interfering with mTORC2 activity, as indicated by AKT (S473) phosphorylation in liver and muscle tissues (Fig. 5H and I). These data suggest that the FDA-approved drug panobinostat inhibits mTORC1 without interfering with mTORC2 activity. As summarized in Fig. S5I, TORSEL is a reporter that reflects the 4EBP1 phosphorylation status balanced by mTORC1 and protein phosphatase (PPase), which results in a switch between diffuse and puncta patterns. Amino acid sensors, such as SESTRIN3, CASTOR, and SAMTOR, and the unknown target “X,” mediate the inhibitory effects of panobinostat on mTORC1.

## Discussion

Basal-level mTORC1 activity is essential for maintaining cellular metabolism under normal culture conditions but is inhibited when organisms are exposed to internal and external stresses such as nutrient starvation, energy depletion, and genotoxic stress. This restriction of mTORC1 activity is critical for cells to maintain metabolic homeostasis autonomously in response to environmental stress and prevent uncontrolled cell proliferation and other pathological damage. Therefore, an inhibition reporter for mTORC1 in living cells could expand the toolbox to facilitate research on mTOR biology.

TORSEL functions similarly to fluorescent protein phase separation-based kinase reporters, such as SPPIER and SPARK [25, 26]. It exhibits a clear and highly contrasted puncta pattern upon mTORC1 inhibition in living cells and in mouse liver tissues, and it possesses several advantages that make it a unique mTORC1 sensor. First, it can effectively report the inhibition of mTORC1 through physiological stress (Fig. 1B and C), pharmacological inhibition (Fig. 1B and D), and genetic perturbation of mTORC1 signaling in living cells (Fig. 2I and Fig. S2E, S2F). Second, TORSEL can be used as a preferential tool for visual screening of mTORC1 inhibitors by imaging a heterogeneous cell population compared to the FRET-based TORCAR reporter (Fig. 2J, Fig. S4A, and Fig. S2J-S2M). Notably, TORSEL is also modulated by protein phosphatases (Fig. 3A and C), and phosphatase activators potentially interfere with the response of TORSEL to mTORC1 inhibition in drug screening; therefore, additional target verification is needed after TORSEL primary screening. Finally, TORSEL effectively responded to mTORC1 inhibition in mouse liver tissues by taking advantage of its high signal/noise ratio and long diffusion time (Fig. 2L, M and N).

By applying the TORSEL reporter to a visual screen in living cells, we successfully identified dozens of drugs that exhibited obvious inhibitory effects on mTORC1. Among them, the HDACis panobinostat and entinostat were validated as mTORC1-specific inhibitors in cell lines and in mouse tissue (Fig. 4B, Fig. S4D, and Fig. 5H and I). Mechanistically, panobinostat induces the expression of nutrient sensor genes, including CASTOR, SAMTOR, and SESTRIN3, through transcriptional regulation. In addition, panobinostat inhibits GATOR1-mediated AA sensing, likely through multiple targets or mechanisms. Notably, the “X” factor in Fig. S5I in the early stage of mTORC1 inhibition before the transcriptional induction of AA sensors is still elusive. One possible mechanism is that panobinostat inhibits HDACs and induces acetylation of non-histone proteins that regulate mTORC1 activation. As previously reported, HDAC6, which deacetylates and stabilizes TSC2 to inhibit mTORC1 [62, 63], could be a target of panobinostat. Indeed, we detected an increase in the TSC2 protein level but not in the mRNA level upon panobinostat treatment (Fig. 5G, Fig. S5B), suggesting that HDAC6 may be involved in panobinostat-induced mTORC1 inhibition through TSC2 acetylation and stability regulation. The clinical benefits of conventional mTOR inhibitors are limited by the relief of feedback inhibition of receptor tyrosine kinase (RTK) signaling, which activates mTORC2-AKT (S473) [64, 65]. Although studies have reported that some HDACis might inhibit mTORC1 by inhibiting AKT (S473) phosphorylation or inducing TSC1 expression [56, 66], none of the HDACi treatments led to evident AKT (S473) inhibition or TSC1 protein changes in our experiments (Figs. 4B and C and 5H and I, and Fig. S4D, S4H). In contrast, we observed a prominent inhibition of GATOR1-mediated AA sensing; for example, panobinostat completely blocked the AA reactivation of mTORC1 but no longer inhibited it in GATOR1-deficient cells upon AA refeeding (Fig. 4D and E). Interestingly, panobinostat still blocks mTORC1 activation through AA signaling in TSC1 knockout cells stimulated with serum (Fig. 4F), suggesting that panobinostat may be useful for treating TSC disease. Whether HDACis inhibit mTORC1 through distinct signaling pathways in a dose-dependent or tissue-specific manner needs detailed verification in animal models and in HDACi-treated clinical samples. Overall, our data will inform further mechanistic dissection and clinical evaluation of HDACis in the treatment of mTORC1-related diseases, especially cancer and aging.

## Conclusions

In summary, our study developed a unique genetically encoded live-cell reporter for mTORC1. Using this reporter, we found that HDACis such as panobinostat inhibit mTORC1 signaling via amino acid sensing targeting, suggesting that panobinostat could be a therapeutic agent for mTOR-related human diseases.

## Materials and methods

### Cell lines and cell treatments

HEK293T (293T), HeLa, U2OS, HCT116, LO2, MCF7, A549, and MEF cells were obtained from ATCC and cultured in DMEM supplemented with 10% FBS, 100 µg/ml streptomycin, and 100 U/ml penicillin. All cells were cultured at 37 °C with 5% CO2. Cells were transfected with polyethylenimine (PEI) or Lipofectamine 3000 according to the manufacturer’s protocols. For serum starvation, the cells were washed twice with PBS and cultured for 12 h in DMEM without FBS. For EAA starvation, cells were cultured in custom-ordered AA-free DMEM supplemented with NEAA overnight, and then EAA was added for stimulation. For whole-AA starvation, cells were cultured in AA-free DMEM for 50 min, and then an EAA and NEAA mixture was added for stimulation. All cell lines were confirmed to be mycoplasma-free before experiments. The cell culture reagents used in this study are listed in Table S6.

### Animal work

Mice were maintained in a pathogen-free environment. For hydrodynamic injection, 6- to 8-week-old BALB/c mice (GemPharmatech Co., Ltd.) were subjected to hydrodynamic tail-vein injections. pmCherry or pmCherry-TORSEL plasmids (40 µg) were diluted in physiological solution (0.9% NaCl) to a volume equivalent to 10% of body weight (0.1 ml/g). The whole volume was intravenously injected quickly, within 5–8 s. After 2 days of recovery, the mice were fasted overnight and intraperitoneally injected with vehicle or AZD8055 (10 mg/kg/per mouse). Two hours later, the mice were euthanized by cervical dislocation, and liver tissues were quickly dissected and stored at -80 °C. Fluorescence images were taken of 5 μm liver cryosections prepared according to a standard protocol. To test the effects of AZD8055 or panobinostat on mTOR signaling in vivo, grouped six-week-old male C57BL/6J mice (GemPharmatech Co., Ltd.) (3–4 mice in each group) were injected intraperitoneally with DMSO, panobinostat (10 mg/kg), or AZD8055 (10 mg/kg) in 30% Captisol. Eight hours postinjection, the mice were euthanized, and the tissues were collected. Liver and muscle tissues were homogenized and lysed with RIPA buffer; total protein was quantified with BCA reagent, and immunoblotting was performed with antibodies as indicated.

### Molecular cloning and plasmids

Nucleic acid fragments of TORSEL or TORCAR were synthesized by Tsingke Biotechnology, China, and subcloned and inserted into the pmCherry vector. All mutants were generated by the standard site-directed mutagenesis method; TORSEL and related mutants were also subcloned and inserted into the pCDH lentiviral vector. Tet-On TORSELs were generated by subcloning TORSEL into the pCW57.1-teton. To generate membranous organelle-targeted reporters, a plasma membrane targeting sequence of the Lyn protein (MGCIKSKRKDK) [17], a mitochondrial targeting sequence of the COX8 protein (MSVLTPLLLRGLTGSARRLPVPRAK) [67], an endoplasmic reticulum targeting sequence of the prolactin signal sequence (MDSKGSSQKGSRLLLLLVVSNLLLCQGVVS) [68], or a lysosomal targeting sequence of the Tmem192 protein [69] were fused to the N-terminus of the reporter. All plasmids were confirmed by Sanger sequencing. The oligos used for subcloning and mutagenesis are listed in Table S6.

### Immunoblotting and immunofluorescence staining

IB and IF experiments were performed as described in [70]; briefly, cells were washed twice with ice-cold PBS and lysed with EBC buffer (50 mM Tris (pH 7.5), 120 mM NaCl, 0.5% NP-40) supplemented with 1 mM dithiothreitol (DTT), protease inhibitors, and phosphatase inhibitors; protein concentrations were measured by Bradford or BCA reagents (Beyotime Biotechnology, China); proteins were resolved by 10 or 12% SDS-PAGE and analyzed by immunoblotting with antibodies as indicated. For IF, the cells were seeded on polylysine-coated glass coverslips and grown overnight; treated as indicated, the cells were fixed with 4% paraformaldehyde (PFA) in PBS for 10 min and permeabilized with 0.1% Triton X-100 in PBS for 5 min. After blocking with 5% bovine serum albumin (BSA), the cells were incubated with primary antibodies and subsequently with anti-rabbit or mouse secondary antibodies conjugated with Alexa Fluor 488 or Alexa Fluor 594. The cells were subsequently stained with DAPI (1 µg/mL) for 5 min, mounted with mounting media (90% glycerol in PBS), and stored at 4 °C until imaging. The primary and secondary antibodies used in this study are listed in Table S6.

### Lentivirus generation, shRNA knockdown, CRISPR/Cas9 gene knockout, and 3xHA endogenous tagging

pLKO.1 lentiviral shRNA virus and pCDH-TORSEL lentiviral virus packaging and subsequent generation of stable cell lines by infection were performed as previously described [70]. Briefly, a 6-well plate of 293T cells was transfected with VSVG, psPAX2, and pLKO.1 shRNA plasmids with PEI. Forty-eight hours posttransfection, the supernatant containing the virus was collected and passed through a 0.45 μm filter, and the virus was stored at − 80 °C until use. For lentiviral transduction, cells were infected overnight with 8 µg/mL polybrene in virus-containing medium, and cells were selected with puromycin for 2 days (48 h) postinfection. For CRISPR/Cas9 gene knockout, individual sgRNAs were subcloned and inserted into LentiCRISPRv2 at the BsmBI site as described in the standard protocol, and dual sgRNAs were subcloned and inserted into LentiCRISPRv2 as described previously [71]. 293T cells were transfected with 2 µg of sgRNA plasmids for each 35-mm dish. Twenty-four hours after transfection, 1 µg/ml puromycin was added to the refreshed medium for 2 days, and puromycin-resistant cells were pooled and amplified for IB analysis. Single-cell clones were isolated using limiting dilution in 96-well plates. For sequencing, genomic DNA was extracted from the clonal cells with lysis buffer (50 mM Tris, pH 8.0, 1 mM EDTA, 0.5% Tween 20, proteinase K > 0.6 U/ml), and amplification of the edited sequence from genomic DNA was performed using Taq PCR mix. The purified PCR products were sequenced to identify the correctly edited clones. For 3xHA endogenous tagging of CASTOR1 in 293T cells, the sgRNA and donor sequences were designed with the online tool TrueDesign Genome (https://apps.thermofisher.cn/apps/genome-editing-portal/#/summary), and construct generation, gene targeting, and 3xHA-tagged CASTOR1 clone identification were performed as previously described [71]. The sgRNA sequences and primers used are listed in Table S6.

### Live-cell microscopic imaging and quantification

The cells were grown in 4-chamber glass-bottom microwell dishes (Cellvis, D35C4-20-1-N). After transfection of TORSEL plasmids as indicated, live-cell imaging was performed with a Nikon ECLIPSE Ti2 inverted microscope with a 60x oil objective. For time-lapse imaging, cells were placed in an incubation chamber maintained at 37 °C with 5% CO2, exposure times were approximately 100 ms for TORSEL, and images were taken at regular intervals. Confocal images were taken by a Zeiss LSM 880 confocal microscope with a 60x oil objective; the exposure times ranged from 50 to 700 ms. To measure the mean fluorescence intensity (MFI) of Tet-On TORSEL, regions of interest (ROIs) were manually defined for each cell in the image. The fluorescence intensity values of the ROIs were then measured in the corresponding channel of background-subtracted images [72]. Cells with saturated fluorescence intensity values were excluded from the analysis. For each of the five randomly selected images, 20–30 cells were analyzed, and the MFI was reported as mean ± SEM. For cellular FRAP experiments, TORSEL puncta induced by rapamycin in U2OS cells were photobleached with a 560 nm laser by Zeiss LSM880 at room temperature. The fluorescence intensities in selected regions were collected every 0.9456 s as the mean ROI, and the mCherry signal values were normalized to the initial intensity before photobleaching. Recovery curves were plotted using GraphPad Prism software. For FRET analysis, cells were cultured in 4-chamber glass-bottom microwell dishes and transfected with TORSEL plasmids as specified. Live-cell imaging was performed using a Zeiss LSM880 inverted microscope equipped with a 40x oil objective. CFP images were acquired using a 405 nm excitation wavelength and an emission range of 450 nm to 521 nm, while YFP images were acquired using a 514 nm excitation wavelength and an emission range of 540 nm to 701 nm. The traces were normalized by setting the emission ratio before drug addition. For quantification of TORSEL-positive cells, three biological replicates were photographed; approximately 50 cells in each sample were calculated by the percentage of TORSEL-positive cells, which is defined by more than 10 visible puncta in one cell area. Based on Fig. 1E, 10 is an effective threshold for discriminating between the background and the responsive TORSEL in these cell lines.

### Compound library screening and data analysis

The screened compounds were stored at a concentration of 10 mM in DMSO. 293T cells stably expressing TORSEL were seeded in 96-well glass bottom plates (P96-1.5P from Cellvis) at 8000 cells per well and grown in complete medium overnight. Plated cells were treated with the test compounds (10 µM), Torin1 (100 nM), or DMSO (as a control) in complete medium for 24 hours. Live-cell imaging was performed by a Nikon ECLIPSE Ti2 inverted microscope with a 60x oil objective as described above. The screening quality was evaluated by the Z’ factor using the following formula:

$${Z}^{{\prime }} factor=1-\frac{3({{\sigma }}_{p}+{{\sigma }}_{n})}{{|{\mu }}_{p}-{{\mu }}_{n}|}$$

µp and σp are the mean and standard deviation values of the positive control (Torin1), respectively, and µn and σn are the mean and standard deviation values of the negative control (DMSO), respectively. For the inhibitor validation experiment, the compounds were separated, and the dose and treatment time are specified in each Figure legend. The library compounds and other inhibitors used in this study are listed in Table S6. For the quantitative analysis of the screening imaging data with ImageJ, the puncta pixel fluorescence intensity and the cell pixel intensity were measured using the “Analyze Particle” function. The puncta signal was first masked to calculate the pixel intensity of the background cells, the background signal was subsequently masked to calculate the puncta signals, and the TORSEL signal was calculated by the following formula:

$$\text{TORSEL\,signal}=\frac{\sum{\text{Puncta}^\prime\text{s}\,\text{pixel\,intensity}}}{\sum{\text{Cel}^\prime\text{s}\,\text{pixel\,intensity}}}$$

To account for variability in the data, the values obtained above were normalized based on the minimum and maximum values, which were used to transform the data into values ranging between 0 and 1. For better accuracy, the positive hits were manually checked because the background signals were not always accurately masked during automatic processing using the ImageJ particle analysis function, and we chose the manual counting method to evaluate the TORSEL response in low-throughput imaging data processing (< 100 images); the negative hits were also normalized to the percentage with Torin1 as the reference, and thresholds were set at 30% to effectively eliminate the noise signals. For automated image data analysis, we used the “Batch-Macro” function in ImageJ.

### RNA-seq data analysis

The sequencing data were filtered with SOAPnuke by (1) removing reads containing sequencing adapters; (2) removing reads whose low-quality base ratio (base quality less than or equal to 15) is more than 20%; (3) removing reads whose unknown base (‘N’ base) ratio is more than 5%. Afterwards, clean reads were obtained and stored in FASTQ format. Bowtie2 was applied to align the clean reads to the gene set, in which known, novel, coding and noncoding transcripts were included. Expression level of gene was calculated by RSEM (v1.3.1).

### RT‒qPCR

Total RNA was extracted with TRIzol according to a standard RNA extraction protocol and dissolved in DEPC-ddH2O. Reverse transcription was performed with 1 µg of total RNA using a 5X Evo M-MLV RT Reaction Mix Ver.2 kit (AG11728, Accurate Biology) according to the manufacturer’s instructions. RT‒qPCR analyses were performed using StepOnePlus with 2× RealStar Fast SYBR qPCR Mix (A303, GenStar). The mRNA levels were quantified with the ΔΔCt method, and the results were normalized to those of β-actin or UBC. All the experiments were performed in triplicate three times. The PCR primers used are listed in Table S6.

### Bioinformatic analysis

The two mRNA expression cohorts were downloaded from the Gene Expression Omnibus (GEO) database under accession numbers GSE191126 and GSE64689. The DEGs between the TSA-treated and untreated groups were identified using the R package “DESeq2”. DEGs were defined using a *P* value less than 0.05 and an absolute log2 (fold change) greater than 0.5.

### Quantification and statistical analysis

ImageJ and GraphPad Prism 9.0 were used to analyze and quantify the data and to plot most of the graphics. The radar plot, bubble plot, scatter plot, and volcano plot were generated with R 4.2.1 (https://www.r-project.org/) (Table S7). Data from biological or technical replicates are shown with the standard error of the mean (SEM). The statistical analysis was performed using a two-tailed Student’s t test, and the *IC50* and *t*_*1/2*_ values were determined by fitting to a standard four-parameter logistic analysis. ^*^P < 0.05 was considered statistically significant. All data from representative experiments were repeated at least three times independently, except those specifically noted in the Figure legends.

### Electronic supplementary material

Below is the link to the electronic supplementary material.

Supplementary Material 1

Supplementary Material 2

## Data Availability

All the data needed to evaluate the conclusions in the paper are presented in the paper and/or the Supplementary Materials. The RNA-seq data have been deposited in the Gene Expression Omnibus (GEO), which is accessible through the GEO accession browser with accession number GSE250539 (https://www.ncbi.nlm.nih.gov/geo/query/acc.cgi?acc=GSE250539). The public microarray datasets with TSA treatments are accessible through the GEO Series accession numbers GSE191126 and GSE64689.

## Abbreviations

TORSEL: mTORC1 sensor for live cells
mTOR: Mammalian or mechanistic target of rapamycin
GF: Growth factor
AA: Amino acids
eIF4E: Eukaryotic initiation factor 4E
4EBP1: eIF4E binding protein 1
S6K: Ribosomal S6 kinase
RAPTOR: Regulatory-associated protein of mTOR
mLST8: Mammalian lethal with SEC 13 protein 8
DEPTOR: DEP domain-containing mTOR-interacting protein
PRAS40: Proline-rich Akt substrate of 40 kDa
GAP: GTPase-activating protein
TSC: Tuberous sclerosis complex
IB: Immunoblotting
IF: Immunofluorescence
IHC: Immunohistochemistry
HDACs: Histone deacetylases
HDACis: Histone deacetylase inhibitors
MOA: Mechanism of action
HOTag3: Homohexameric tag 3
HOTag6: Homotetrameric tag 6
Dc: Degree of clustering
FRAP: Fluorescence recovery after photobleaching
