# Supplementary material for: TORSEL, a 4EBP1-based mTORC1 live-cell sensor, reveals nutrient-sensing targeting by histone deacetylase inhibitors
Source: Cell Biosci. 2024 Jun 1;14:68. doi: 10.1186/s13578-024-01250-4 (PMC11143692; doi:10.1186/s13578-024-01250-4)
Supplement: Supplementary file 1 — Supplementary Material 1 [file 13578_2024_1250_MOESM1_ESM.docx]

**Additional file 1: Supplementary figures and figure legends**

**
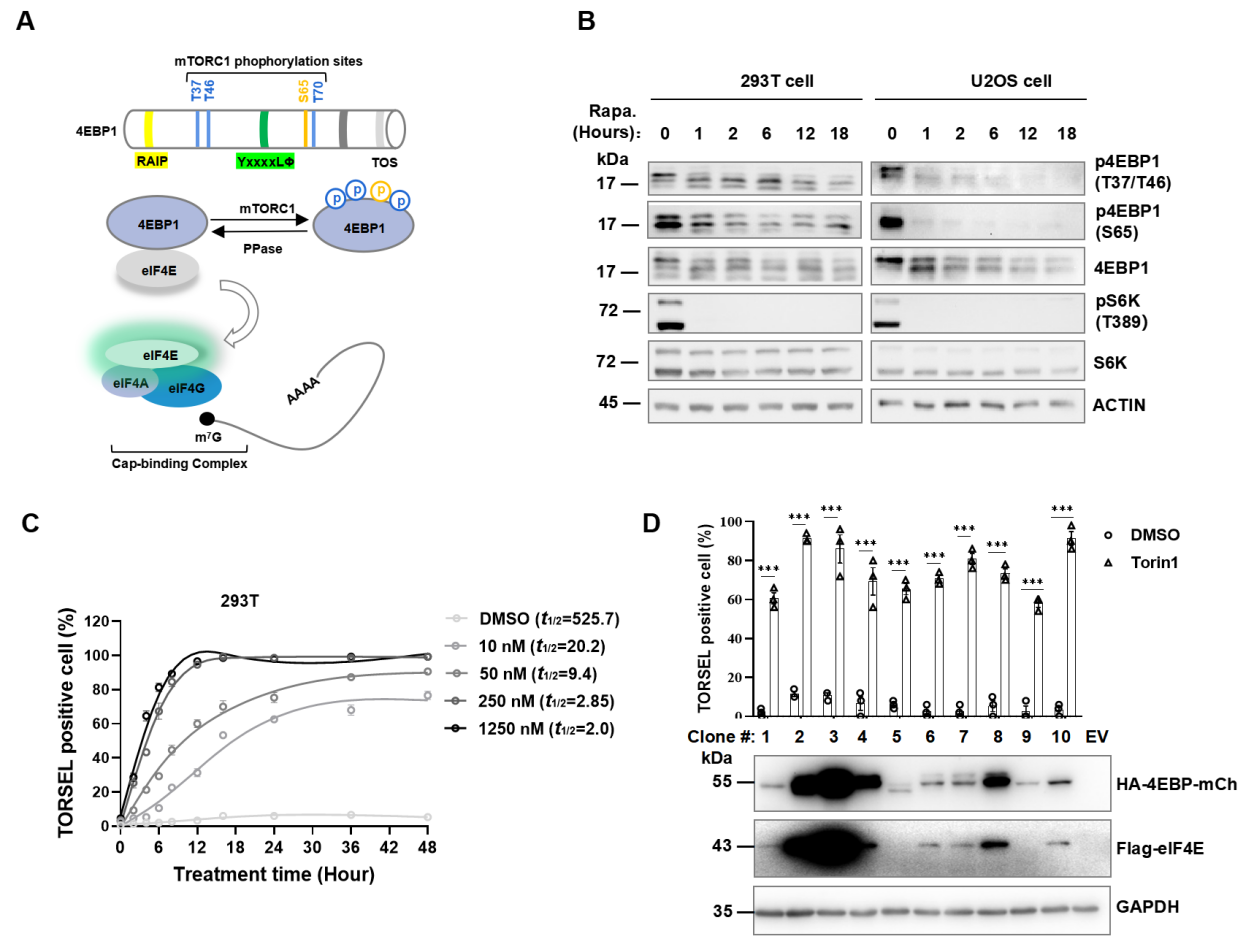
**

**Fig. S1 Additional characterization of TORSEL.**

1. Diagram depicting the regulation of the cap-binding complex by mTORC1-mediated 4EBP1 phosphorylation in mRNA translation initiation.
2. Time-course IB analysis of 4EBP1 phosphorylation in 293T and U2OS cells treated with rapamycin (100 nM) for the indicated times.
3. Time-response curve of TORSEL for different doses of Torin1 in 293T cells. The *t*_1/2_ for each dose was calculated with nonlinear fit analysis, 3 replicates at each time point were calculated.
4. IB analysis of stable 293T clones expressing different levels of TORSEL (lower panel), and responses of TORSEL in stable 293T clones to Torin1 inhibition (50 nM, 12 hours) (upper panel).

Data are presented as the mean ± SEM. Approximately 50 cells in each sample were calculated by percentage of TORSEL-positive cells. Statistical analysis was performed using two-tailed unpaired Student’s t-test. ^✱^*P* < 0.05, ^✱✱^*P* < 0.01, ^✱✱✱^*P* < 0.001.

**
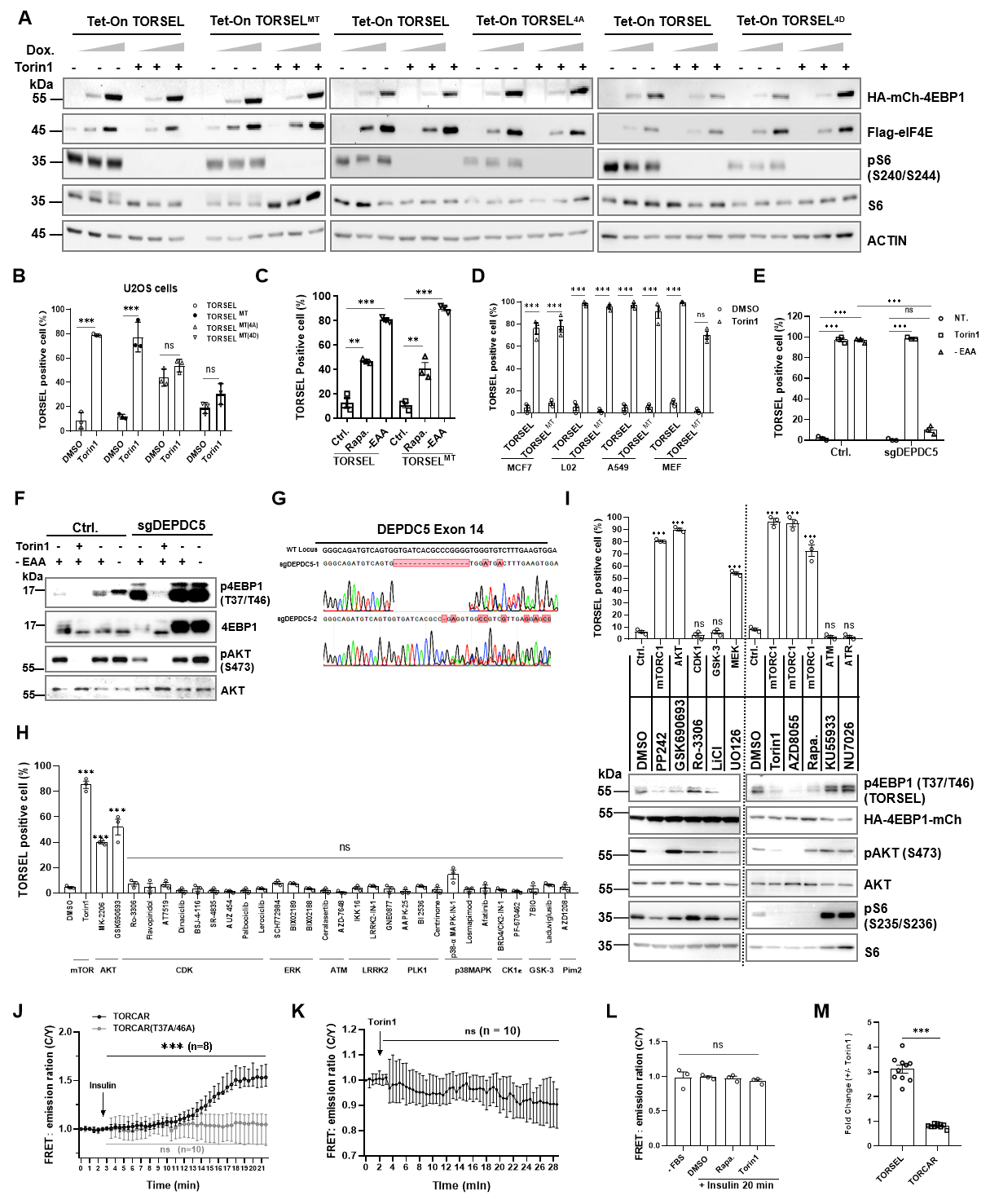
**

**Fig. S2 TORSEL and TORSEL^MT^ respond to mTORC1-mediated 4EBP1 phosphorylation.**

1. IB analysis of Tet-On TORSELs induced by Dox (0.01, 0.05, and 1.0 µg/ml) in 293T cells treated with Torin1 (50 nM, 12 hours).
2. Responses of TORSELs to Torin1 in U2OS cells.
3. Responses of TORSELs to EAA starvation and rapamycin (100 nM, 12 hours) in U2OS cells.
4. Responses of TORSELs to Torin1 in MEFs and various human cancer cell lines.
5. Quantified TORSEL responses to EAA starvation in DEPDC5-KO 293T cells treated with Torin1 or EAA starvation for 12 hours.
6. IB analysis of mTORC1 signaling in wild-type and DEPDC5-KO 293T cells.
7. Sequencing results of PCR products amplified from the CRISPR/Cas9 edited DEPDC5 genomic locus.
8. Responses of TORSEL to various inhibitors targeting 4EBP1 kinases from the kinase library screening in Fig. 2J.
9. Comparison of the effects of inhibitors by TORSEL reporter and IB analysis. 293T cells were treated for 12 hours with PP242 (100 nM ), GSK690693 (100 nM), Ro-3306 (1.0 μM), LiCl (10 mM), U0126 (10 μM), Torin1 (50 nM), AZD8055 (100 nM), rapamycin (100 nM), KU55933 (2 μM), or NU7026 (10 μM).
10. Time course response of TORCAR to insulin (100 nM). 293T cells expressing TORCAR (n = 8) or TORCAR-T37A/46A (n = 10) were serum-starved and then stimulated with insulin, and the normalized FRET emission signals (CFP/YPET) were compared.
11. Time course analysis of the TORCAR response to Torin1 by microscopy in 293T cells. FRET signals under basal conditions, and after Torin1 addition were compared for 30 min (n=10 cells).
12. TORCAR response to insulin (100 nM) with mTOR pre-inhibition in 96-well plate reading mode. The peak FRET signals at 20 min of treatment were compared.
13. Comparison of the TORSEL Dc value and TORCAR FRET signal fold change in 10 single cells treated with Torin1 treatments in 293T cells.

The Data are presented as the mean ± SEM. Statistical analysis was performed using two-tailed unpaired Student’s t-test.ns, no statistical significance, ^✱^*P* < 0.05, ^✱✱^*P* < 0.01, ^✱✱✱^*P* < 0.001.


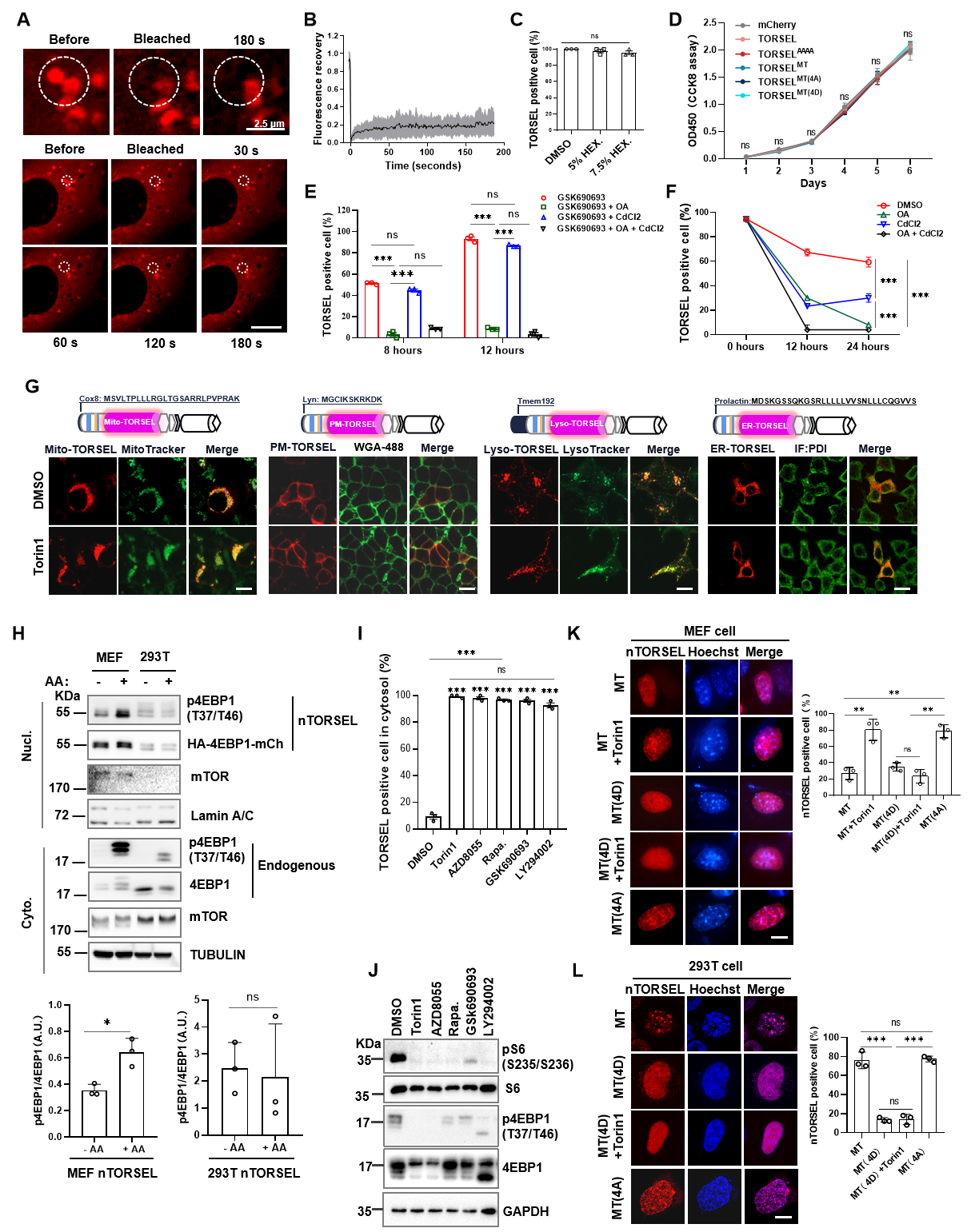


**Fig. S3 Regulation of TORSEL by protein phosphatases.**

1. Representative images of TORSEL puncta in U2OS cells. After bleaching (circled area), time-lapse images were taken at the indicated intervals for 3 min.
2. Quantified FRAP signals for TORSEL puncta. (n = 10).
3. The effect of HEX on TORSEL in 293T cells. TORSEL puncta were induced by Torin1, then treated with HEX for 10 min, TORSEL-positive cells were calculated.
4. Cell proliferation analysis of 293T cells stably expressing mCherry, TORSEL, TORSEL^AAAA^, TORSEL^MT^, TORSEL^MT(4A)^, or TORSEL^MT(4D)^.
5. Effects of phosphatase inhibitors on TORSEL’s responses induced by GSK690693. OA (20 nM), CdCl (25.0 μM), and GSK690693 (100 nM).
6. Time-course effects of OA, CdCl2, or both on the diffusion of TORSEL puncta after GSK690693 washing out.
7. The responses of membranous organelle-targeted TORSEL to Torin1. The response of Mito-TORSEL, PM-TORSEL, Lyso-TORSEL, and ER-TORSEL to Torin1 (50 nM,12 hours) in 293T cells. The mitochondria, plasma membrane, lysosomes were labeled with their tracker dyes, and the endoplasmic reticulum was probed with PDI.

Data are presented as the mean ± SEM. Statistical analysis was performed using two-tailed unpaired Student’s t-test. ns, no statistical significance, ^✱^*P* < 0.05, ^✱✱^*P* < 0.01, ^✱✱✱^*P* < 0.001. Scale bar, 10 μm.

**
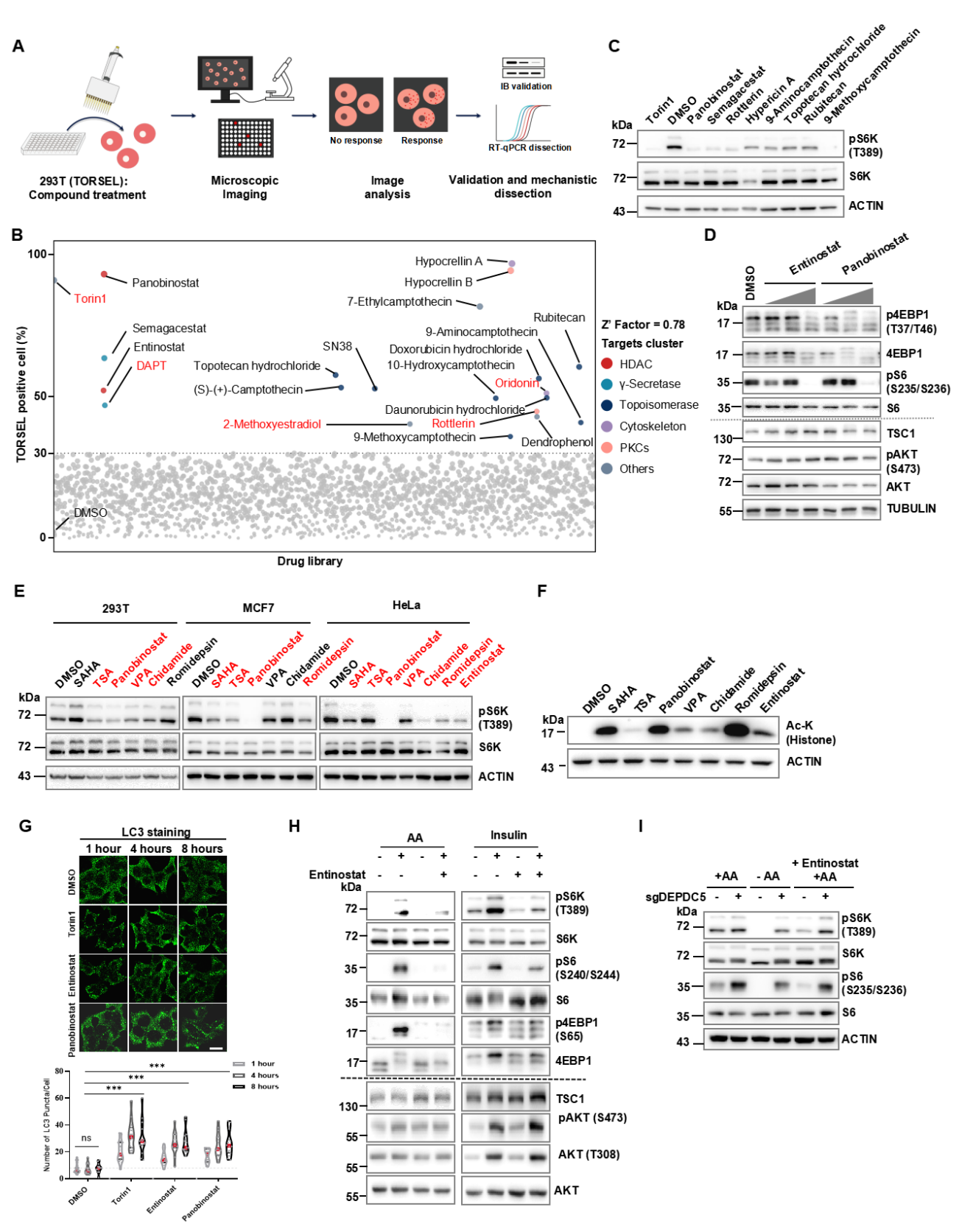
**

**Fig. S4 TORSEL high-throughput screening identified HDACis as mTORC1 inhibitors.**

1. Flow diagram for live-cell screening of TORSEL-expressing 293T cells.
2. Scatter plots of drug library screening by TORSEL. The red-highlighted drugs are known mTORC1 inhibitors, the gray dots indicate no or weak inhibitory activity, the colored dots indicate the grouped hits, and the dotted line shows the 30% threshold for screening.
3. IB validation of mTORC1 activity in response to the positive hits in 293T cells.
4. IB analysis of mTORC1 signaling in MCF7 cells treated with dose escalation (0.1, 1.0, or 10 μM for 12 hours) treatment with HDACis.
5. IB analysis of mTORC1 activity after treatment with HDACis (1.0 μM SAHA, 1.0 μM TSA, 10 μM panobinostat, 1.0 μM VPA, 1.0 μM chidamide, 1.0 μM romidepsin, and 10 μM entinostat). mTORC1-inhibiting drugs are highlighted in red.
6. IB analysis of acetylated histones in response to HDACis.
7. Autophagy analysis by LC3 A/B staining in response to Torin1 (50 nM), entinostat (10 μM), or panobinostat (10 μM) (left panel). Violin plots of quantified LC3 A/B puncta per cell, red line, median; black line, interquartile ranges, 15-20 cells were quantified from each of three biological replicates.(right panel).
8. IB analysis of mTOR activity in response to entinostat. 293T cells were pretreated with DMSO or 10 μM entinostat for 12 hours, starved with AA for 50 min and then stimulated with AA for 15 min, or serum starved for 12 hours and stimulated with 100 nM insulin for 15 min.
9. IB analysis of mTORC1 activity with 10 μM entinostat inhibition in DEPDC5-depleted 293T cells with or without AA stimulation.

Data are presented as the mean ± SEM. Statistical analysis was performed with two-tailed unpaired Student’s t-test. ns, no statistical significance, ^✱^*P* < 0.05, ^✱✱^*P* < 0.01, ^✱✱✱^*P* < 0.001. Scale bar, 10 μm.

**
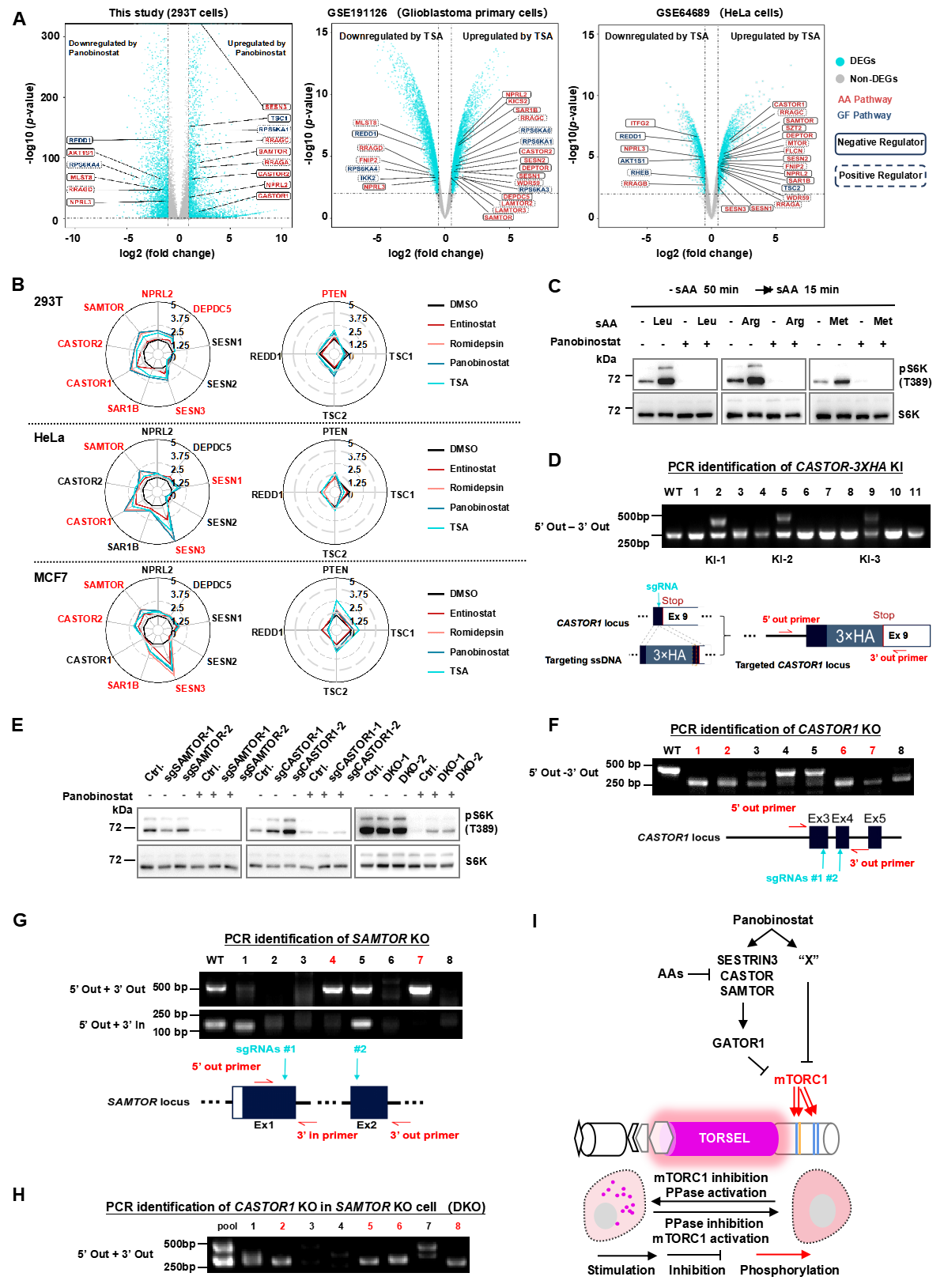
**

**Fig. S5. Transcription induction of AA sensors contributes to mTORC1 inhibition by HDACis.**

1. Volcano plot depicting DEGs from three HDACi-treated transcriptomic datasets. Colored dots represent positive hits with a P value <0.05 and a log2 fold change > 0.5. The DEGs were grouped by the AA pathway (red) and the GF pathway (blue) and marked as the negative (solid box) or positive (dashed box) regulators of mTORC1.
2. Radar plots of mRNA levels in response to HDACi treatments. Relative mRNA levels of major mTORC1-inhibitory genes involved in the AA pathway and the GF pathway were quantified using RT-qPCR in 293T, HeLa, and MCF7 cells treated with four types of HDACis for 12 hours.
3. IB analysis of panobinostat-mediated inhibition of mTORC1. Single AA (sAA) starved for 2 hours, followed by 15 min of sAA stimulation in 293T cells. Leu (400 μM), Arg (600 μM) and Met (100 μM).
4. The identification strategy for 3xHA endogenously tagged CASTOR1 gene loci, correctly targeted loci were identified by out-out primers flanking the last exon; the upper band PCR product showed the 3xHA tagged locus, and the lower band showed the wild-type locus.
5. IB analysis of mTORC1 inhibition by panobinostat (10 μM, 12 hours) in SAMTOR, CASTOR1, or double knockout (DKO) 293T cells; the experiment was repeated twice.

(F)-(H) PCR identification of SAMTOR or CASTOR1 gene knockout or DKO via the two-sgRNA strategy in 293T clonal cells.

(I) Schematic diagram of the TORSEL reporter and the inhibition mechanisms of panobinostat. The TORSEL reporter is regulated by mTORC1 activity and protein phosphatase (PPase), which results in a switch between diffuse and puncta patterns. Amino acid sensors, such as SESTRIN3, CASTOR, and SAMTOR, or targets "X" mediate the inhibitory effects of panobinostat on mTORC1 through amino acid sensing or other mechanisms.
