# Supplementary material for: TORSEL, a 4EBP1-based mTORC1 live-cell sensor, reveals nutrient-sensing targeting by histone deacetylase inhibitors
Source: Cell Biosci. 2024 Jun 1;14:68. doi: 10.1186/s13578-024-01250-4 (PMC11143692; doi:10.1186/s13578-024-01250-4)
Supplement: Supplementary file 2 — Supplementary Material 2 [file 13578_2024_1250_MOESM2_ESM.docx]

**Additional file 2: Supplementary tables**

**Table S1 Amino acid sequences of TORSEL and TORSEL mutants, related to Figure 2**

|  | HA-4EBP1-mCherry-Linker-HOTag3-P2A-Flag-eIF4E-Linker-HOTag6 |
| --- | --- |
| **TORSEL** | MYPYDVPDYAMSGGSSCSQTPSRAIPATRRVVLGDGVQLPPGDYSTTPGGTLFSTTPGGTRIIYDRKFLMECRNSPVTKTPPRDLPTIPGVTSPSSDEPPMEASQSHLRNSPEDKRAGGEESQFEMDIGGSGSGGGTPVATMVSKGEEDNMAIIKEFMRFKVHMEGSVNGHEFEIEGEGEGRPYEGTQTAKLKVTKGGPLPFAWDILSPQFMYGSKAYVKHPADIPDYLKLSFPEGFKWERVMNFEDGGVVTVTQDSSLQDGEFIYKVKLRGTNFPSDGPVMQKKTMGWEASSERMYPEDGALKGEIKQRLKLKDGGHYDAEVKTTYKAKKPVQLPGAYNVNIKLDITSHNEDYTIVEQYERAEGRHSTGGMDELYKSGLRSGSGSAGGSAGGSAGGSAGGSAGGSAGGSAGGSRGEIAKSLKEIAKSLKEIAWSLKEIAKSLKGSGATNFSLLKQAGDVEENPGPKLDYKDDDDKMATVEPETTPTPNPPTTEEEKTESNQEVANPEHYIKHPLQNRWALWFFKNDKSKTWQANLRLISKFDTVEDFWALYNHIQLSSNLMPGCDYSLFKDGIEPMWEDEKNKRGGRWLITLNKQQRRSDLDRFWLETLLCLIGESFDDYSDDVCGAVVNVRAKGDKIAIWTTECENREAVTHIGRVYKERLGLPPKIVIGYQSHADTATKSGSTTKNRFVVVDGSGSAGGSAGGSAGGSAGGSAGGSAGGSAGGSRTLREIEELLRKIIEDSVRSVAELEDIEKWLKKI* |
| TORSEL4A | MYPYDVPDYAMSGGSSCSQTPSRAIPATRRVVLGDGVQLPPGDYSTAPGGTLFSTAPGGTRIIYDRKFLMECRNAPVTKAPPRDLPTIPGVTSPSSDEPPMEASQSHLRNSPEDKRAGGEESQFEMDIGGSGSGGGTPVATMVSKGEEDNMAIIKEFMRFKVHMEGSVNGHEFEIEGEGEGRPYEGTQTAKLKVTKGGPLPFAWDILSPQFMYGSKAYVKHPADIPDYLKLSFPEGFKWERVMNFEDGGVVTVTQDSSLQDGEFIYKVKLRGTNFPSDGPVMQKKTMGWEASSERMYPEDGALKGEIKQRLKLKDGGHYDAEVKTTYKAKKPVQLPGAYNVNIKLDITSHNEDYTIVEQYERAEGRHSTGGMDELYKSGLRSGSGSAGGSAGGSAGGSAGGSAGGSAGGSAGGSRGEIAKSLKEIAKSLKEIAWSLKEIAKSLKGSGATNFSLLKQAGDVEENPGPKLDYKDDDDKMATVEPETTPTPNPPTTEEEKTESNQEVANPEHYIKHPLQNRWALWFFKNDKSKTWQANLRLISKFDTVEDFWALYNHIQLSSNLMPGCDYSLFKDGIEPMWEDEKNKRGGRWLITLNKQQRRSDLDRFWLETLLCLIGESFDDYSDDVCGAVVNVRAKGDKIAIWTTECENREAVTHIGRVYKERLGLPPKIVIGYQSHADTATKSGSTTKNRFVVVDGSGSAGGSAGGSAGGSAGGSAGGSAGGSAGGSRTLREIEELLRKIIEDSVRSVAELEDIEKWLKKI* |
| TORSEL4D | MYPYDVPDYAMSGGSSCSQTPSRAIPATRRVVLGDGVQLPPGDYSTDPGGTLFSTDPGGTRIIYDRKFLMECRNDPVTKDPPRDLPTIPGVTSPSSDEPPMEASQSHLRNSPEDKRAGGEESQFEMDIGGSGSGGGTPVATMVSKGEEDNMAIIKEFMRFKVHMEGSVNGHEFEIEGEGEGRPYEGTQTAKLKVTKGGPLPFAWDILSPQFMYGSKAYVKHPADIPDYLKLSFPEGFKWERVMNFEDGGVVTVTQDSSLQDGEFIYKVKLRGTNFPSDGPVMQKKTMGWEASSERMYPEDGALKGEIKQRLKLKDGGHYDAEVKTTYKAKKPVQLPGAYNVNIKLDITSHNEDYTIVEQYERAEGRHSTGGMDELYKSGLRSGSGSAGGSAGGSAGGSAGGSAGGSAGGSAGGSRGEIAKSLKEIAKSLKEIAWSLKEIAKSLKGSGATNFSLLKQAGDVEENPGPKLDYKDDDDKMATVEPETTPTPNPPTTEEEKTESNQEVANPEHYIKHPLQNRWALWFFKNDKSKTWQANLRLISKFDTVEDFWALYNHIQLSSNLMPGCDYSLFKDGIEPMWEDEKNKRGGRWLITLNKQQRRSDLDRFWLETLLCLIGESFDDYSDDVCGAVVNVRAKGDKIAIWTTECENREAVTHIGRVYKERLGLPPKIVIGYQSHADTATKSGSTTKNRFVVVDGSGSAGGSAGGSAGGSAGGSAGGSAGGSAGGSRTLREIEELLRKIIEDSVRSVAELEDIEKWLKKI* |
| TORSELMT | MYPYDVPDYAMAGGAACAQAPARAIPAARRVVLGDGVQLPPGDYAATPGGALFAATPGGARIIYDRKFLMECRNSPVAKTPPRDLPAIPGVAAPAADEPPMEAAQAHLRNAPEDKRAGGEEAQFEMDIGGSGSGGGTPVATMVSKGEEDNMAIIKEFMRFKVHMEGSVNGHEFEIEGEGEGRPYEGTQTAKLKVTKGGPLPFAWDILSPQFMYGSKAYVKHPADIPDYLKLSFPEGFKWERVMNFEDGGVVTVTQDSSLQDGEFIYKVKLRGTNFPSDGPVMQKKTMGWEASSERMYPEDGALKGEIKQRLKLKDGGHYDAEVKTTYKAKKPVQLPGAYNVNIKLDITSHNEDYTIVEQYERAEGRHSTGGMDELYKSGLRSGSGSAGGSAGGSAGGSAGGSAGGSAGGSAGGSRGEIAKSLKEIAKSLKEIAWSLKEIAKSLKGSGATNFSLLKQAGDVEENPGPKLDYKDDDDKMATVEPETTPTPNPPTTEEEKTESNQEVANPEHYIKHPLQNRWALWFFKNDKSKTWQANLRLISKFDTVEDFWALYNHIQLSSNLMPGCDYSLFKDGIEPMWEDEKNKRGGRWLITLNKQQRRSDLDRFWLETLLCLIGESFDDYSDDVCGAVVNVRAKGDKIAIWTTECENREAVTHIGRVYKERLGLPPKIVIGYQSHADTATKSGSTTKNRFVVVDGSGSAGGSAGGSAGGSAGGSAGGSAGGSAGGSRTLREIEELLRKIIEDSVRSVAELEDIEKWLKKI* |
| TORSELMT(4A) | MYPYDVPDYAMAGGAACAQAPARAIPAARRVVLGDGVQLPPGDYAAAPGGALFAAAPGGARIIYDRKFLMECRNAPVAKAPPRDLPAIPGVAAPAADEPPMEAAQAHLRNAPEDKRAGGEEAQFEMDIGGSGSGGGTPVATMVSKGEEDNMAIIKEFMRFKVHMEGSVNGHEFEIEGEGEGRPYEGTQTAKLKVTKGGPLPFAWDILSPQFMYGSKAYVKHPADIPDYLKLSFPEGFKWERVMNFEDGGVVTVTQDSSLQDGEFIYKVKLRGTNFPSDGPVMQKKTMGWEASSERMYPEDGALKGEIKQRLKLKDGGHYDAEVKTTYKAKKPVQLPGAYNVNIKLDITSHNEDYTIVEQYERAEGRHSTGGMDELYKSGLRSGSGSAGGSAGGSAGGSAGGSAGGSAGGSAGGSRGEIAKSLKEIAKSLKEIAWSLKEIAKSLKGSGATNFSLLKQAGDVEENPGPKLDYKDDDDKMATVEPETTPTPNPPTTEEEKTESNQEVANPEHYIKHPLQNRWALWFFKNDKSKTWQANLRLISKFDTVEDFWALYNHIQLSSNLMPGCDYSLFKDGIEPMWEDEKNKRGGRWLITLNKQQRRSDLDRFWLETLLCLIGESFDDYSDDVCGAVVNVRAKGDKIAIWTTECENREAVTHIGRVYKERLGLPPKIVIGYQSHADTATKSGSTTKNRFVVVDGSGSAGGSAGGSAGGSAGGSAGGSAGGSAGGSRTLREIEELLRKIIEDSVRSVAELEDIEKWLKKI* |
| TORSELMT(4D) | MYPYDVPDYAMAGGAACAQAPARAIPAARRVVLGDGVQLPPGDYAADPGGALFAADPGGARIIYDRKFLMECRNDPVAKDPPRDLPAIPGVAAPAADEPPMEAAQAHLRNAPEDKRAGGEEAQFEMDIGGSGSGGGTPVATMVSKGEEDNMAIIKEFMRFKVHMEGSVNGHEFEIEGEGEGRPYEGTQTAKLKVTKGGPLPFAWDILSPQFMYGSKAYVKHPADIPDYLKLSFPEGFKWERVMNFEDGGVVTVTQDSSLQDGEFIYKVKLRGTNFPSDGPVMQKKTMGWEASSERMYPEDGALKGEIKQRLKLKDGGHYDAEVKTTYKAKKPVQLPGAYNVNIKLDITSHNEDYTIVEQYERAEGRHSTGGMDELYKSGLRSGSGSAGGSAGGSAGGSAGGSAGGSAGGSAGGSRGEIAKSLKEIAKSLKEIAWSLKEIAKSLKGSGATNFSLLKQAGDVEENPGPKLDYKDDDDKMATVEPETTPTPNPPTTEEEKTESNQEVANPEHYIKHPLQNRWALWFFKNDKSKTWQANLRLISKFDTVEDFWALYNHIQLSSNLMPGCDYSLFKDGIEPMWEDEKNKRGGRWLITLNKQQRRSDLDRFWLETLLCLIGESFDDYSDDVCGAVVNVRAKGDKIAIWTTECENREAVTHIGRVYKERLGLPPKIVIGYQSHADTATKSGSTTKNRFVVVDGSGSAGGSAGGSAGGSAGGSAGGSAGGSAGGSRTLREIEELLRKIIEDSVRSVAELEDIEKWLKKI* |

**Table S2 Information on Kinase inhibitors grouped by target signaling pathway, related to Figure 2**

| **Target signaling pathway** | **Number of inhibitors** |
| --- | --- |
| Cell Cycle | 59 |
| GPCR/G Protein | 4 |
| JAK/STAT Signaling | 7 |
| MAPK/ERK Pathway | 28 |
| Membrane Transporter/Ion Channel | 3 |
| NF-κB | 4 |
| PI3K/Akt/mTOR | 9 |
| Protein Tyrosine Kinase/RTK | 61 |
| Stem Cell/Wnt | 20 |
| TGF-β/Smad | 8 |
| Other unclassified | 48 |

**Table S3 Putative 4EBP1 kinases in specific phosphorylation context with literature reference, related to Figure 2**

| **Kinase name** | **Phosphorylation-sites** | **Dependence on mTOR** | **Phosphorylation context** | **Reference** |
| --- | --- | --- | --- | --- |
| AKT | ND | Yes | GF stimulation | [1, 2] |
| ATM | S111 | ND | GF stimulation | [3] |
| Pim2 | T37/T46/S65/T70 | ND | GF stimulation | [4] |
| ERK1/2 | S65/T70 | Yes | TPA stimulated | [5] |
| CDK1 | T37/T46/S65/S83/S101 | ND | Cell cycle-dependant | [6-9] |
| CDK4 | T37/T46/S65/S101 | ND | Cell cycle-dependant | [10] |
| CDK12 | S65/T70 | Yes | Cell cycle-dependant | [11] |
| PLK1 | T37/T46 | ND | Paclitaxel induced cell cycle arrest | [12, 13] |
| p38/MSK | T37/T46/S65/T70 | ND | UVB induced DNA Damage | [14] |
| LRRK2 | T37/T46 | ND | Oxidative stress | [15] |
| CK1ε | T41/T50 | ND | Specified in breast cancer cell lines | [16] |
| GSK3β | T37/T46/S6/Thr 70 | ND | Specified in some cancer cell lines | [17] |

ND, not determined

**Table S4 Information on positive hits from compound screening, related to Figure 2 and 4**

| **Inhibitor** | **Target** | **Relation to mTOR** | **Reference** | **Punctate cell (%)** |
| --- | --- | --- | --- | --- |
| Buparlisib | PI3K | PI3K/AKT/mTOR pathway | [18, 19] | 97.06197 |
| Torin1 | mTOR | PI3K/AKT/mTOR pathway | [20] | 85.3 |
| GSK690693 | AKT/AMPK | PI3K/AKT/mTOR pathway | [21, 22] | 60 |
| PP121 | mTOR/PDGFR | PI3K/AKT/mTOR pathway | [23] | 49.84095 |
| MK-2206 | AKT | PI3K/AKT/mTOR pathway | [24] | 39.68765 |
| CCT128930 | AKT | PI3K/AKT/mTOR pathway | [25] | 38.37867 |
| GDC-0575 | CHK1 | Genotoxic stress inhibit mTORC1 | [26] | 55 |
| WNK463 | WNKs | mTOR regulating kinase | [27] | 64.46429 |
| SKI-178 | SPHK | mTOR regulating kinase | [28, 29] | 38.33333 |
| Entinostat | HDAC | ND | ND | 52.08333 |
| panobinostat | HDAC | ND | ND | 93.09524 |
| Semagacestat | Ƴ-secretase | ND | ND | 63.47882 |
| DAPT | Ƴ-Secretase | mTORC1 inhibition | [30] | 46.82821 |
| 2-Methoxyestradiol | microtubule/HIF-1α/2α | mTORC1 inhibition | [31] | 40.24123 |
| Rottlerin | PKC | mTORC1 inhibition | [32-34] | 44.67019 |
| Oridonin | AKT | mTORC1 inhibition | [35] | 51.07415 |
| Dendrophenol | NF-κB | ND | ND | 42.80423 |
| Hypocrellin B | Unknown | Autofluorescence/ND | [36] | 94.3105 |
| Hypocrellin A | PKC/others | Autofluorescence/ND | [36] | 96.82111 |
| Topotecan hydrochloride | Topoisomerase I | mTORC1 inhibition | [26] | 57.51355 |
| 10-Hydroxycamptothecin | Topoisomerase I | mTORC1 inhibition | [26] | 48.18713 |
| (S)-(+)-Camptothecin | Topoisomerase I | mTORC1 inhibition | [26] | 53.09106 |
| SN38 | Topoisomerase I | mTORC1 inhibition | [26] | 52.73268 |
| Hydroxy Camptothecine | Topoisomerase I | mTORC1 inhibition | [26] | 49.80495 |
| 7-Ethylcamptothecin | Topoisomerase I | mTORC1 inhibition | [26] | 81.66667 |
| 10-Hydroxycamptothecin | Topoisomerase I | mTORC1 inhibition | [26] | 49.32921 |
| 9-Methoxycamptothecin | Topoisomerase I | mTORC1 inhibition | [26] | 35.83057 |
| Doxorubicin hydrochloride | Topoisomerase I | mTORC1 inhibition | [26] | 56.28816 |
| Daunorubicin hydrochloride | Topoisomerase II | mTORC1 inhibition | [26] | 49.45773 |
| Rubitecan | Topoisomerase I | mTORC1 inhibition | [26] | 60.401 |
| 9-Aminocamptothecin | Topoisomerase I | mTORC1 inhibition | [26] | 40.76479 |

**Table S5 Eighty-nine genes selected from the mTOR KEGG pathway, related to Figure 5**

| **Gene** | **Complex** | **Effect on mTORC1** | **pathway** |
| --- | --- | --- | --- |
| *MTOR* | mTORC1 | Positive | AA/GF |
| *RPTOR* | mTORC1 | Positive | AA/GF |
| *MLST8* | mTORC1 | Positive | AA/GF |
| *AKT1S1* | mTORC1 | Negative | AA/GF |
| *DEPTOR* | mTORC1 | Negative | AA/GF |
| *CASTOR1* | CASTOR | Negative | AA |
| *CASTOR2* | CASTOR | Negative | AA |
| *BMT2* | SAMTOR | Negative | AA |
| *SESN1* | SESTRIN | Negative | AA |
| *SESN2* | SESTRIN | Negative | AA |
| *SESN3* | SESTRIN | Negative | AA |
| *SAR1B* | SAR1B | Negative | AA |
| *NPRL2* | GATOR1 | Negative | AA |
| *NPRL3* | GATOR1 | Negative | AA |
| *DEPDC5* | GATOR1 | Negative | AA |
| *KPTN* | KICSTOR | Negative | AA |
| *ITFG2* | KICSTOR | Negative | AA |
| *KICS2* | KICSTOR | Negative | AA |
| *SZT2* | KICSTOR | Negative | AA |
| *MIOS* | GATOR2 | Positive | AA |
| *SEC13* | GATOR2 | Positive | AA |
| *WDR59* | GATOR2 | Positive | AA |
| *SEH1L* | GATOR2 | Positive | AA |
| *WDR24* | GATOR2 | Positive | AA |
| *RRAGA* | Rag | Positive | AA |
| *RRAGB* | Rag | Positive | AA |
| *RRAGC* | Rag | Positive | AA |
| *RRAGD* | Rag | Positive | AA |
| *LAMTOR1* | Ragulator | Positive | AA |
| *LAMTOR2* | Ragulator | Positive | AA |
| *LAMTOR3* | Ragulator | Positive | AA |
| *LAMTOR4* | Ragulator | Positive | AA |
| *LAMTOR5* | Ragulator | Positive | AA |
| *SLC38A9* | SLC38A9/SNAT9 | Positive | AA |
| *FLCN* | FLCN/FNIP | Positive | AA |
| *FNIP2* | FLCN/FNIP | Positive | AA |
| *ATP6V1G3* | v-ATPase | Positive | AA |
| *ATP6V1C2* | v-ATPase | Positive | AA |
| *ATP6V1D* | v-ATPase | Positive | AA |
| *ATP6V1H* | v-ATPase | Positive | AA |
| *ATP6V1A* | v-ATPase | Positive | AA |
| *ATP6V1B1* | v-ATPase | Positive | AA |
| *ATP6V1B2* | v-ATPase | Positive | AA |
| *ATP6V1C1* | v-ATPase | Positive | AA |
| *ATP6V1E1* | v-ATPase | Positive | AA |
| *ATP6V1G2* | v-ATPase | Positive | AA |
| *ATP6V1E2* | v-ATPase | Positive | AA |
| *ATP6V1F* | v-ATPase | Positive | AA |
| *ATP6V1G1* | v-ATPase | Positive | AA |
| *ATP6V0A1* | v-ATPase | Positive | AA |
| *LARS1* | LARS1 | Positive | AA |
| *TSC1* | TSC | Negative | GF |
| *TSC2* | TSC | Negative | GF |
| *TBC1D7* | TSC | Negative | GF |
| *DDIT4* | REDD1 | Negative | GF |
| *PRKAA1* | AMPK | Negative | GF |
| *PRKAA2* | AMPK | Negative | GF |
| *PRKAB1* | AMPK | Negative | GF |
| *PRKAB2* | AMPK | Negative | GF |
| *PRKAG1* | AMPK | Negative | GF |
| *PRKAG2* | AMPK | Negative | GF |
| *PRKAG3* | AMPK | Negative | GF |
| *RPS6KB1* | S6K1 | Negative | GF |
| *RPS6KB2* | S6K2 | Negative | GF |
| *CHUK* | TNF | Negative | GF |
| *IKBKB* | TNF | Negative | GF |
| *GSK3B* | WNT | Negative | GF |
| *PTEN* | PTEN | Negative | GF |
| *GRB10* | GRB10 | Negative | GF |
| *IRS1* | IRS | Positive | GF |
| *PDPK1* | PDK1 | Positive | GF |
| *PIK3CA* | PI3K | Positive | GF |
| *PIK3CB* | PI3K | Positive | GF |
| *PIK3CG* | PI3K | Positive | GF |
| *PIK3CD* | PI3K | Positive | GF |
| *PIK3R1* | PI3K | Positive | GF |
| *PIK3R2* | PI3K | Positive | GF |
| *AKT1* | AKT | Positive | GF |
| *AKT2* | AKT | Positive | GF |
| *AKT3* | AKT | Positive | GF |
| *MAPK1* | ERK | Positive | GF |
| *MAPK3* | ERK | Positive | GF |
| *RPS6KA1* | RSK | Positive | GF |
| *RPS6KA2* | RSK | Positive | GF |
| *RPS6KA3* | RSK | Positive | GF |
| *RPS6KA4* | RSK | Positive | GF |
| *RPS6KA5* | RSK | Positive | GF |
| *RPS6KA6* | RSK | Positive | GF |
| *RHEB* | Rheb | Positive | GF |

| **Table S6 Key reagents for this study** | | | |
| --- | --- | --- | --- |
| **Reagent name** | **Source** | **Catalog number** |  |
| **Primary antibodies** | | |  |
| anti-S6 | BETHYL | A300-557A |  |
| anti-pS6(S235/S236) | Cell Signaling Technology | 4858S |  |
| anti-pS6(S240/S244) | Solarbio | K006232P |  |
| anti-PanAKT | Abclonal | A3145 |  |
| anti-pAKT1(S473) | Cell Signaling Technology | 4060S |  |
| anti-pAKT1(T308) | Cell Signaling Technology | 4056S |  |
| anti-LC3 A/B | Cell Signaling Technology | 12741S |  |
| anti-4EBP1 | Cell Signaling Technology | 9644 |  |
| anti-p4EBP1(T37/46) | Cell Signaling Technology | 2855 |  |
| anti-p4EBP1(S65) | Cell Signaling Technology | 9451S |  |
| anti-TSC1 | Cell Signaling Technology | 6935 |  |
| anti-HA | Cell Signaling Technology | 3724 |  |
| anti-Flag | Sigma-Aldrich | F7425 |  |
| anti-mTOR | Santa Cruz | sc-1549 |  |
| anti-mTOR | Cell Signaling Technology | 2983T |  |
| anti-Raptor | Cell Signaling Technology | 2280T |  |
| anti-Rictor | BETHYL | A300-459A |  |
| anti-S6K | Cell Signaling Technology | 2708 |  |
| anti-pS6K(T389) | Cell Signaling Technology | 9234 |  |
| anti-GAPDH | Sangon Biotech | AB10016 |  |
| anti-Tubulin | Invitrogen | 236-10501 |  |
| anti-LaminA/C | Cell Signaling Technology | 4777 |  |
| anti-LAMP2 | Santa Cruz | sc-377385 |  |
| anti-SESN3 | Proteintech | 11431-2-AP |  |
| anti-TSC2 | HUABIO | ET1610 |  |
| anti-PRAS40 | HUABIO | HA721581 |  |
| anti-GβL | Cell Signaling Technology | 3274 |  |
| anti-PDI | Cell Signaling Technology | 2446 |  |
| **Secondary antibody** | | |  |
| HRP-labeled Goat Anti-Mouse IgG(H+L） | Beyotime | A0216 |  |
| HRP-labeled Goat Anti-Rabbit IgG(H+L) | Beyotime | A0208 |  |
| Goat Anti-Mouse IgG H&L (Alexa Fluor® 594) | abcam | ab150116 |  |
| Goat Anti-Rabbit IgG H&L (Alexa Fluor® 488) | abcam | ab150077 |  |
| **Compound and inhibitors** | | |  |
| Torin1 | Selleck | S2827 |  |
| Rapamycin | Selleck | S1039 |  |
| AZD8055 | TargetMol | T1859 |  |
| Wortmannin | TargetMol | T6283 |  |
| GSK690693 | Selleck | T6285 |  |
| PP242 | Selleck | T2414 |  |
| LiCl | TargetMol | T15633 |  |
| CdCl2 | Aladdin | C116344 |  |
| KU55933 | TargetMol | T2685 |  |
| NU7026 | TargetMol | T2433 |  |
| Ro3306 | TargetMol | T2356 |  |
| U0126 | TargetMol | T21332 |  |
| LY294002 | TargetMol | T2008 |  |
| Entinostat | APExBIO | A8171 |  |
| Panobinostat | APExBIO | A8178 |  |
| Semagacestat | APExBIO | A8190 |  |
| DAPT | APExBIO | A8200 |  |
| Rottlerin | TargetMol | T16791 |  |
| Hypericin | TargetMol | T6S0923 |  |
| 9-Aminocamptothecin | TargetMol | T3S1957 |  |
| Topotecan hydrochloride | TargetMol | T1174 |  |
| Rubitecan | TargetMol | T5853 |  |
| 9-Methoxycamptothecin | TargetMol | T5S1952 |  |
| SAHA | Selleck | S1047 |  |
| TSA | Selleck | T6270 |  |
| VPA | Selleck | S3944 |  |
| Chidamide | Selleck | S8567 |  |
| Romidepsin | Beyotime | SD1087 |  |
| Insulin | YEASEN | 40112ES25 |  |
| 1,6-Hexanedoil | Sigma | 240117 |  |
| Captisol | Aladdin | C125030 |  |
| COmplete™ Protease Inhibitor | Roche | 4693132001 |  |
| Phosphatase inhibitor cocktail A | Beyotime | P1081 |  |
| L-Leucine | Sigma | L8912 |  |
| L-Argine HCl | Amresco | 877 |  |
| L-Methionine | Amresco | E801 |  |
| Lyso Tracker Green DND-26 | Cell Signaling Technology | 8783 |  |
| Mito Tracker Green FM | Cell Signaling Technology | 9074 |  |
| Wheat Germ Agglutinin (WGA) | Invitrogen | W7024 |  |
| Doxycycline hyclate | Beyotime | ST039A |  |
| **Drug library** | | |  |
| DiscoveryProbe™ FDA-approved Drug Library | APExBIO | L1021 |  |
| Anti-Tumor Natural Product Library | TargetMol | L6700 |  |
| Kinase inhibitor library | MedChemExpress | Customer ordered |  |
| **Cell culture and transfection reagents** | | |  |
| DMEM | Gibco | C11995500BT |  |
| AA free DMEM | BasalMedia | X014C |  |
| FBS | ExCell | FSP500 |  |
| MEM Amino Acids (100 X EAA) | Gibco | 11130077 |  |
| MEM Non-Essential Amino Acids (100X NEAA) | Gibco | 11140050 |  |
| Streptomycin and penicillin | Gibco | 15140122 |  |
| PEI | Sigma | 408727 |  |
| Lipofectamine 3000 | Invitrogen | L3000008 |  |
| Puromycin | Beyotime | ST551 |  |
| Hygromycin | Beyotime | ST1389 |  |
| **Cell lines** | | |  |
| HEK293T | ATCC | CRL-3216 |  |
| HeLa | ATCC | N/A |  |
| MCF7 | ATCC | N/A |  |
| HCT116 | ATCC | N/A |  |
| LO2 | ATCC | N/A |  |
| A549 | ATCC | N/A |  |
| MEF | Lab stock | N/A |  |
| U2OS | Lab stock | N/A |  |
| **Primers used in quantitative RT‒PCR** | | |  |
| **Gene** | **Primer** | **Sequence (5'-3')** |  |
| *NPRL2* | Forward | AAAGAAGCTGATCGGCTGTCC |  |
|  | Reverse | GCTCTCTAGCTCTAGTGTGGT |  |
| *DEPDC5* | Forward | AGTGTTCCGGCTGAGACCTTA |  |
|  | Reverse | CCACGGCCAATATACTGATCCT |  |
| *SESN1* | Forward | TGCTTTGGGCCGTTTGGATAA |  |
|  | Reverse | TGTAGTGACGATAATGTAGGGGT |  |
| *SESN2* | Forward | TCTTACCTGGTAGGCTCCCAC |  |
|  | Reverse | AGCAACTTGTTGATCTCGCTG |  |
| *SESN3* | Forward | ACCTGCTCTGTACCAACTGC |  |
|  | Reverse | GACGACCGGATGTAGAGTATTCT |  |
| *SAR1B* | Forward | TACAGTGGTTTCAGCAGTGTG |  |
|  | Reverse | AGTGGGATGTAATGTTGGGACA |  |
| *CASTOR1* | Forward | TCGCCACCACCCTCATAGAT |  |
|  | Reverse | AGGTCACTGGGGAACTTTTTCT |  |
| *CASTOR2* | Forward | AGGAAGGATTCCTAGAGCTGC |  |
|  | Reverse | AGTGGGGCGATGACTGACTT |  |
| *SAMTOR* | Forward | AGAAGTACCGAGAAGTGGGAG |  |
|  | Reverse | ACCTTCGCCCTCACAAGTTT |  |
| *PTEN* | Forward | TTTGAAGACCATAACCCACCAC |  |
|  | Reverse | ATTACACCAGTTCGTCCCTTTC |  |
| *TSC1* | Forward | CAACAAGCAAATGTCGGGGAG |  |
|  | Reverse | CATAGGGCCACGGTCAGAA |  |
| *TSC2* | Forward | ATAGCTGTTACCTCGACGAGT |  |
|  | Reverse | TGCAGGGAGACCTCTATGTCC |  |
| *REDD1* | Forward | TGGGCAAAGAACTACTGCG |  |
|  | Reverse | AGAGTTGGCGGAGCTAAACAG |  |
| *ACTB* | Forward | CATGTACGTTGCTATCCAGGC |  |
|  | Reverse | CTCCTTAATGTCACGCACGAT |  |
| *UBC* | Forward | ATTTGGGTCGCGGTTCTTG |  |
|  | Reverse | TGCCTTGACATTCTCGATGGT |  |
| **sgRNA sequences** | | |  |
| **Gene** | **Primer** | **Sequence (5'-3')** |  |
| sgDEPDC5-1 | Forward | CACCGGTCAGTGGTGATCACGCCCG |  |
|  | Reverse | AAACCGGGCGTGATCACCACTGACC |  |
| sgDEPDC5-2 | Forward | CACCGTGTTAATGTCGTAGACCCTA |  |
|  | Reverse | AAACTAGGGTCTACGACATTAACA |  |
| sgTSC1-1 | Foward | CACCGGGAGCATGTGCGAATTCATC |  |
|  | Reverse | AAACGATGAATTCGCACATGCTCCC |  |
| sgTSC1-2 | Foward | CACCGCGTGGCGGAAGTCTATCTCG |  |
|  | Reverse | AAACCGAGATAGACTTCCGCCACGC |  |
| sgCASTOR1- | Forward | ACGCGTCTCACACCGGACACGTGGTGCTCGGCCAGGTTTTAGAGCTAGAAATAGCAAG |  |
| (Dual-sgRNA primer) |  |  |  |
|  | Reverse | ACGCGTCTCAAAACGCGTGTGGATCACCACGGACCGGTGTTTCGTCCTTTCCAC |  |
| sgSAMTOR- | Forward | ACGCGTCTCACACCGGGTACTTCTTGCGGAGCCGCGTTTTAGAGCTAGAAATAGCAAG |  |
| (Dual-sgRNA primer) |  |  |  |
|  | Reverse | ACGCGTCTCAAAACCACAGTGTTCTCGCCAGATCCGGTGTTTCGTCCTTTCCAC |  |
| sgCASTOR1 (KI) | Forward | CACCGGTCCTCCAGCGGCGGCAGGA |  |
|  | Reverse | AAACTCCTGCCGCCGCTGGAGGACC |  |
| **Primers used in point mutation and subcloning** | | |  |
| **Gene** | **Primer** | **Sequences (5'-3')** |  |
| *4EBP1* | Forward | GGTTAAGCTTGACTACAAGGACGATGACGA |  |
|  | Reverse | GGTTACCGGTGTGCCTCCACCGCTGCCAGATCCGCCAATGTCCATCTCAAACTGTG |  |
| *4EBP1*(S65A/T70A) | Forward | ATGGAGTGTCGGAACGCCCCTGTGACCAAAGCCCCCCCAAGGGATCTG |  |
|  | Reverse | CAGATCCCTTGGGGGGGCTTTGGTCACAGGGGCGTTCCGACACTCCAT |  |
| *4EBP1*(S65D/T70D) | Forward | CTGATGGAGTGTCGGAACGACCCTGTGACCAAAGACCCCCCAAGGGATCTG |  |
|  | Reverse | CAGATCCCTTGGGGGGTCTTTGGTCACAGGGTCGTTCCGACACTCCATCAG |  |
| *4EBP1*(T37A/T46A) | Forward | GGGGACTACAGCACGGCCCCCGGCGGCACGCTCTTCAGCACCGCCCCGGGAGGTACCAGG |  |
|  | Reverse | CCTGGTACCTCCCGGGGCGGTGCTGAAGAGCGTGCCGCCGGGGGCCGTGCTGTAGTCCCC |  |
| *4EBP1*(T37D/T46D) | Forward | GGGGACTACAGCACGGACCCCGGCGGCACGCTCTTCAGCACCGACCCGGGAGGTACCAGG |  |
|  | Reverse | CCTGGTACCTCCCGGGTCGGTGCTGAAGAGCGTGCCGCCGGGGTCCGTGCTGTAGTCCCC |  |
| *4EBP1(MT-*T36A/T46A) | Forward | GGGGACTACGCCGCGGCACCCGGCGGCGCGCTCTTCGCCGCAGCACCGGGAGGTGCCAGG |  |
|  | Reverse | CCTGGCACCTCCCGGTGCTGCGGCGAAGAGCGCGCCGCCGGGTGCCGCGGCGTAGTCCCC |  |
| *4EBP1(MT-*T37D/T46D) | Forward | GGGGACTACGCCGCGGACCCCGGCGGCGCGCTCTTCGCCGCAGACCCGGGAGGTGCCAG |  |
|  | Reverse | CTGGCACCTCCCGGGTCTGCGGCGAAGAGCGCGCCGCCGGGGTCCGCGGCGTAGTCCCC |  |
| *4EBP1(MT-*S65A/T70A) | Forward | ATGGAGTGTCGGAACGCCCCTGTGGCCAAAGCACCCCCAAGGGATCTG |  |
|  | Reverse | CAGATCCCTTGGGGGTGCTTTGGCCACAGGGGCGTTCCGACACTCCAT |  |
| *4EBP1(MT-*S65D/T70D) | Forward | ATGGAGTGTCGGAACGATCCTGTGGCCAAAGACCCCCCAAGGGATCTG |  |
|  | Reverse | CAGATCCCTTGGGGGGTCTTTGGCCACAGGATCGTTCCGACACTCCAT |  |
| *4EBP1(YLAA)* | Forward | CGGGAGGTACCAGGATCATCGCTGACCGGAAATTCGCGATGGAGTGTCGGAACTCACC |  |
|  | Reverse | GGTGAGTTCCGACACTCCATCGCGAATTTCCGGTCAGCGATGATCCTGGTACCTCCCG |  |
| *4EBP1(AAAA)* | Forward | GCTGCAGCCAGACCCCAAGCGCGGCCGCCGCCGCCACTCGCCGGGTGGTGC |  |
|  | Reverse | GCACCACCCGGCGAGTGGCGGCGGCGGCCGCGCTTGGGGTCTGGCTGCAGC |  |
| *PM-4EBP1* | Forward | CGCTAGCGCCACCATGGGCTGCATCAAGAGCAAGCGCAAGGACAAGTACCCTTATGATG |  |
|  | Reverse | CAAGCTTTTGTCCTTGCGCTTGCTCTTGATGCAGCCCATTGGGCCAGGATTCTCCTCGA |  |
| *Mito-4EBP1* | Forward-F1 | CCTCTAGAGCCACCATGGAGCTCATGTCCGTCCTGACGCCGCTGCTGCTGC |  |
|  | Forward-F2 | CCTGACGCCGCTGCTGCTGCGGGGCTTGACAGGCTCGGCCCGGCGGCTCC |  |
|  | Reverse | AGCTAGCCTTGGCGCGCGGCACTGGGAGCCGCCGGGCCGAGCCTG |  |
| *Tmem192* | Forward | ATTCTAGAGCCACCATGGCGGCGG |  |
|  | Reverse | AGCTAGCCGTTCTACTTGGCTGACAGCCC |  |
| *ER-4EBP1* | Forward-F1 | CTCTAGAGCCACCATGGAGCTCATGGACAGCAAAGGTTCGTCGCAGAAAGGGTCC |  |
|  | Forward-F2 | GTTCGTCGCAGAAAGGGTCCCGCCTGCTCCTGCTGCTGGTGGTGTCAAATCTACT |  |
|  | Reverse | TAGCTAGCGGAGACCACACCCTGGCACAAGAGTAGATTTGACACCACCAG |  |
| **Primers used in genomic PCR and PCR product sequencing** | | |  |
| **Gene** | **Primer** | **Sequence (5'-3')** |  |
| *DEPDC5* allele PCR | Forward | GGCTGCACAGGGCAAGTATG |  |
|  | Reverse | GGGGAAATCAGGCTAGGAACA |  |
| *CASTOR1* allele PCR | 5’ out | TGCCCTTACCTTGATGGAGC |  |
|  | 3’ out | CCCAAGTCCAAGTGTCAGGG |  |
| *SAMTOR* allele PCR | 5’ out | GTGTGGTGAAGAGCGTCCAC |  |
|  | 3’ out | TGGCACTATGAATTATACACAGCA |  |
|  | 3’ in | GGGACGTGCAGATCCTTTACATTT |  |
| *CASTOR1* KI | Forward | AGGAGGTAGTCTCAGCTGGG |  |
|  | Reverse | GTACACAGGTGTCCGCGTAA |  |
| **Mouse** | | |  |
| BALB/c mice | GemPharmatech Co.,Ltd., Guangzhou | https://www.gempharmatech.com/ |  |
| C57BL/6 mice | GemPharmatech Co.,Ltd. Guangzhou | https://www.gempharmatech.com/ |  |
| **Plasmid vectors** |  |  |  |
| lentiCRISPR v2 | Addgene | 52961 |  |
| pCDH-CMV | Addgene | 72265 |  |
| pCW57.1 Tet-ON | Addgene | 41393 |  |
| pmCherry | Lab stock | N/A |  |
| pLKO.1 | Lab stock | N/A |  |
|  |  |  |  |

| ImageJ | National Institutes of Health | https://imagej.nih.gov/ij |
| --- | --- | --- |
| GraphPad Prism 8.0 | GraphPad Software | https://www.graphpad.com |
| R 4.2.1 | Online R Project for Statistical Computing | https://www.r-project.org/ |
| DESeq2 | Online Bioconductor software | https://bioconductor.org/ |
|  |  |  |

**Table S7 Software and algorithms used for this study**

**References**

1. Anne-Claude Gingras SGK, Maura A. O’Leary, Nahum Sonenberg, and Nissim Hay 4E-BP1, a repressor of mRNA translation, is phosphorylated and inactivated by the Akt(PKB) signaling pathway .pdf. *GENES & DEVELOPMENT* 1997, 12(0890-9369/98 ):502–513.

2. Gingras AC, Kennedy SG, O'Leary MA, Sonenberg N, Hay N: 4E-BP1, a repressor of mRNA translation, is phosphorylated and inactivated by the Akt(PKB) signaling pathway. *Genes & Development* 1998, 12(4):502-513.

3. Yang DQ, Kastan MB: Participation of ATM in insulin signalling through phosphorylation of eIF-4E-binding protein 1. *Nat Cell Biol* 2000, 2(12):893-898.

4. Fox CJ, Hammerman PS, Cinalli RM, Master SR, Chodosh LA, Thompson CB: The serine/threonine kinase Pim-2 is a transcriptionally regulated apoptotic inhibitor. *Genes Dev* 2003, 17(15):1841-1854.

5. Herbert TP, Tee AR, Proud CG: The extracellular signal-regulated kinase pathway regulates the phosphorylation of 4E-BP1 at multiple sites. *Journal of Biological Chemistry* 2002, 277(13):11591-11596.

6. Greenberg VL, Zimmer SG: Paclitaxel induces the phosphorylation of the eukaryotic translation initiation factor 4E-binding protein 1 through a Cdk1-dependent mechanism. *Oncogene* 2005, 24(30):4851-4860.

7. Shuda M, Velasquez C, Cheng E, Cordek DG, Kwun HJ, Chang Y, Moore PS: CDK1 substitutes for mTOR kinase to activate mitotic cap-dependent protein translation. *Proc Natl Acad Sci U S A* 2015, 112(19):5875-5882.

8. Celestino Velásquez EC, Masahiro Shuda, Paula J. Lee-Oesterreich, Lisa Pogge von Strandmann, Marina A. Gritsenko, Jon M. Jacobs, Patrick S. Moore, and Yuan Chang: Mitotic protein kinase CDK1 phosphorylation of mRNA translation regulator 4E-BP1 Ser83 may contribute to cell transformation. pdf. *PNAS* 2016, 113:8466–8471.

9. Velasquez C, Cheng E, Shuda M, Lee-Oesterreich PJ, Pogge von Strandmann L, Gritsenko MA, Jacobs JM, Moore PS, Chang Y: Mitotic protein kinase CDK1 phosphorylation of mRNA translation regulator 4E-BP1 Ser83 may contribute to cell transformation. *Proc Natl Acad Sci U S A* 2016, 113(30):8466-8471.

10. Mitchell DC, Menon A, Garner AL: Cyclin-dependent kinase 4 inhibits the translational repressor 4E-BP1 to promote cap-dependent translation during mitosis-G1 transition. *FEBS Lett* 2020, 594(8):1307-1318.

11. Choi SH, Martinez TF, Kim S, Donaldson C, Shokhirev MN, Saghatelian A, Jones KA: CDK12 phosphorylates 4E-BP1 to enable mTORC1-dependent translation and mitotic genome stability. *Genes Dev* 2019, 33(7-8):418-435.

12. Shang ZF, Yu L, Li B, Tu WZ, Wang Y, Liu XD, Guan H, Huang B, Rang WQ, Zhou PK: 4E-BP1 participates in maintaining spindle integrity and genomic stability via interacting with PLK1. *Cell Cycle* 2012, 11(18):3463-3471.

13. Severance AL, Latham KE: PLK1 regulates spindle association of phosphorylated eukaryotic translation initiation factor 4E-binding protein and spindle function in mouse oocytes. *Am J Physiol Cell Physiol* 2017, 313(5):C501-C515.

14. Liu G, Zhang Y, Bode AM, Ma WY, Dong Z: Phosphorylation of 4E-BP1 is mediated by the p38/MSK1 pathway in response to UVB irradiation. *J Biol Chem* 2002, 277(11):8810-8816.

15. Imai Y, Gehrke S, Wang HQ, Takahashi R, Hasegawa K, Oota E, Lu B: Phosphorylation of 4E-BP by LRRK2 affects the maintenance of dopaminergic neurons in Drosophila. *EMBO J* 2008, 27(18):2432-2443.

16. Shin S, Wolgamott L, Roux PP, Yoon SO: Casein kinase 1epsilon promotes cell proliferation by regulating mRNA translation. *Cancer Res* 2014, 74(1):201-211.

17. Shin S, Wolgamott L, Tcherkezian J, Vallabhapurapu S, Yu Y, Roux PP, Yoon SO: Glycogen synthase kinase-3 beta positively regulates protein synthesis and cell proliferation through the regulation of translation initiation factor 4E-binding protein 1. *Oncogene* 2014, 33(13):1690-1699.

18. Burger MT, Pecchi S, Wagman A, Ni ZJ, Knapp M, Hendrickson T, Atallah G, Pfister K, Zhang Y, Bartulis S *et al*: Identification of NVP-BKM120 as a Potent, Selective, Orally Bioavailable Class I PI3 Kinase Inhibitor for Treating Cancer. *ACS Med Chem Lett* 2011, 2(10):774-779.

19. McPherson V, Reardon B, Bhayankara A, Scott SN, Boyd ME, Garcia-Grossman IR, Regazzi AM, McCoy AS, Kim PH, Al-Ahmadie H *et al*: A phase 2 trial of buparlisib in patients with platinum-resistant metastatic urothelial carcinoma. *Cancer* 2020, 126(20):4532-4544.

20. Thoreen CC, Kang SA, Chang JW, Liu Q, Zhang J, Gao Y, Reichling LJ, Sim T, Sabatini DM, Gray NS: An ATP-competitive mammalian target of rapamycin inhibitor reveals rapamycin-resistant functions of mTORC1. *J Biol Chem* 2009, 284(12):8023-8032.

21. Rhodes N, Heerding DA, Duckett DR, Eberwein DJ, Knick VB, Lansing TJ, McConnell RT, Gilmer TM, Zhang SY, Robell K *et al*: Characterization of an Akt kinase inhibitor with potent pharmacodynamic and antitumor activity. *Cancer Res* 2008, 68(7):2366-2374.

22. Altomare DA, Zhang L, Deng J, Di Cristofano A, Klein-Szanto AJ, Kumar R, Testa JR: GSK690693 delays tumor onset and progression in genetically defined mouse models expressing activated Akt. *Clin Cancer Res* 2010, 16(2):486-496.

23. Apsel B, Blair JA, Gonzalez B, Nazif TM, Feldman ME, Aizenstein B, Hoffman R, Williams RL, Shokat KM, Knight ZA: Targeted polypharmacology: discovery of dual inhibitors of tyrosine and phosphoinositide kinases. *Nat Chem Biol* 2008, 4(11):691-699.

24. Hirai H, Sootome H, Nakatsuru Y, Miyama K, Taguchi S, Tsujioka K, Ueno Y, Hatch H, Majumder PK, Pan BS *et al*: MK-2206, an allosteric Akt inhibitor, enhances antitumor efficacy by standard chemotherapeutic agents or molecular targeted drugs in vitro and in vivo. *Mol Cancer Ther* 2010, 9(7):1956-1967.

25. Yap TA, Walton MI, Hunter LJ, Valenti M, de Haven Brandon A, Eve PD, Ruddle R, Heaton SP, Henley A, Pickard L *et al*: Preclinical pharmacology, antitumor activity, and development of pharmacodynamic markers for the novel, potent AKT inhibitor CCT128930. *Mol Cancer Ther* 2011, 10(2):360-371.

26. Ma Y, Vassetzky Y, Dokudovskaya S: mTORC1 pathway in DNA damage response. *Biochim Biophys Acta Mol Cell Res* 2018, 1865(9):1293-1311.

27. Liu Z, Demian W, Persaud A, Jiang C, Subramanaya AR, Rotin D: Regulation of the p38-MAPK pathway by hyperosmolarity and by WNK kinases. *Sci Rep* 2022, 12(1):14480.

28. Kim MH, Park JW, Lee EJ, Kim S, Shin SH, Ahn JH, Jung Y, Park I, Park WJ: C16‑ceramide and sphingosine 1‑phosphate/S1PR2 have opposite effects on cell growth through mTOR signaling pathway regulation. *Oncol Rep* 2018, 40(5):2977-2987.

29. Jesko H, Stepien A, Lukiw WJ, Strosznajder RP: The Cross-Talk Between Sphingolipids and Insulin-Like Growth Factor Signaling: Significance for Aging and Neurodegeneration. *Mol Neurobiol* 2019, 56(5):3501-3521.

30. Song BQ, Chi Y, Li X, Du WJ, Han ZB, Tian JJ, Li JJ, Chen F, Wu HH, Han LX *et al*: Inhibition of Notch Signaling Promotes the Adipogenic Differentiation of Mesenchymal Stem Cells Through Autophagy Activation and PTEN-PI3K/AKT/mTOR Pathway. *Cell Physiol Biochem* 2015, 36(5):1991-2002.

31. Zhou X, Liu C, Lu J, Zhu L, Li M: 2-Methoxyestradiol inhibits hypoxia-induced scleroderma fibroblast collagen synthesis by phosphatidylinositol 3-kinase/Akt/mTOR signalling. *Rheumatology (Oxford)* 2018, 57(9):1675-1684.

32. Balgi AD, Fonseca BD, Donohue E, Tsang TC, Lajoie P, Proud CG, Nabi IR, Roberge M: Screen for chemical modulators of autophagy reveals novel therapeutic inhibitors of mTORC1 signaling. *PLoS One* 2009, 4(9):e7124.

33. Torricelli C, Daveri E, Salvadori S, Valacchi G, Ietta F, Muscettola M, Carlucci F, Maioli E: Phosphorylation-independent mTORC1 inhibition by the autophagy inducer Rottlerin. *Cancer Lett* 2015, 360(1):17-27.

34. Daveri E, Maellaro E, Valacchi G, Ietta F, Muscettola M, Maioli E: Inhibitions of mTORC1 and 4EBP-1 are key events orchestrated by Rottlerin in SK-Mel-28 cell killing. *Cancer Lett* 2016, 380(1):106-113.

35. Wang YY, Lv YF, Lu L, Cai L: Oridonin inhibits mTOR signaling and the growth of lung cancer tumors. *Anticancer Drugs* 2014, 25(10):1192-1200.

36. Xu S, Zhang X, Chen S, Zhang M, Shen T: The fluorescence properties of hypocrellin B and its amino-substituted derivative: photoinduced intramolecular proton transfer and photoinduced intramolecular electron transfer. *Photochem Photobiol* 2004, 80:112-114.
